# Structural Studies on Diverse Betacyanin Classes in Matured Pigment-Rich Fruits of *Basella alba* L. and *Basella alba* L. var. ‘Rubra’ (Malabar Spinach)

**DOI:** 10.3390/ijms231911243

**Published:** 2022-09-24

**Authors:** Katarzyna Sutor-Świeży, Michał Antonik, Ewa Dziedzic, Monika Bieniasz, Przemysław Mielczarek, Łukasz Popenda, Karol Pasternak, Małgorzata Tyszka-Czochara, Sławomir Wybraniec

**Affiliations:** 1 Department C-1, Faculty of Chemical Engineering and Technology, Cracow University of Technology, ul. Warszawska 24, 31-155 Krakow, Poland; 2 Faculty of Biotechnology and Horticulture, Agricultural University of Krakow, al. 29 Listopada 54, 31-425 Krakow, Poland; 3 Department of Analytical Chemistry and Biochemistry, Faculty of Materials Science and Ceramics, AGH University of Science and Technology, al. Adama Mickiewicza 30, 30-059, Krakow, Poland; 4 Laboratory of Proteomics and Mass Spectrometry, Maj Institute of Pharmacology, Polish Academy of Sciences, ul. Smętna 12, 31-343 Krakow, Poland; 5 NanoBioMedical Centre, Adam Mickiewicz University, ul. Wszechnicy Piastowskiej 3, 61-614 Poznan, Poland; 6 Institute of Bioorganic Chemistry, Polish Academy of Sciences, ul. Noskowskiego 12/14, 61-704 Poznan, Poland; 7 Faculty of Pharmacy, Jagiellonian University Medical College, ul. Medyczna 9, 30-688 Krakow, Poland

**Keywords:** nitrogenous acyl moiety, gomphrenins, betacyanins, betalains, betanidin, *Basella alba*, *Basella alba* var. ‘Rubra’, Malabar spinach, plant pigments, NMR

## Abstract

Identification of betacyanins in *Basella alba* L. and *Basella alba* L. var. ‘Rubra’ fruits was performed by low- and high-resolution mass spectrometry (LRMS and HRMS) as well as ^1^H, ^13^C and two-dimensional NMR which revealed hitherto completely not known betacyanin classes in the plant kingdom. Especially, the presence of unique nitrogenous acyl moieties in the structures of the pigments was ascertained by the HRMS Orbitrap detection. Except for detected polar betacyanin glycosylated derivatives, presence of a series of previously not reported pigments such as malonylated betanidin 6-*O*-*β*-glusosides with their acyl migration isomers along with the evidence of the 3′′-hydroxy-butyrylated betacyanins is reported. The first complete NMR data were obtained for novel and principal acylated gomphrenins with hydroxycinnamic acids: 6′-*O*-*E*-caffeoyl-gomphrenin (malabarin), 6′-*O*-*E*-sinapoyl-gomphrenin (gandolin), 6′-*O*-*E*-4-coumaroyl-gomphrenin (globosin) and 6′-*O*-*E*-feruloyl-gomphrenin (basellin).

## 1. Introduction

**Figure 1:**
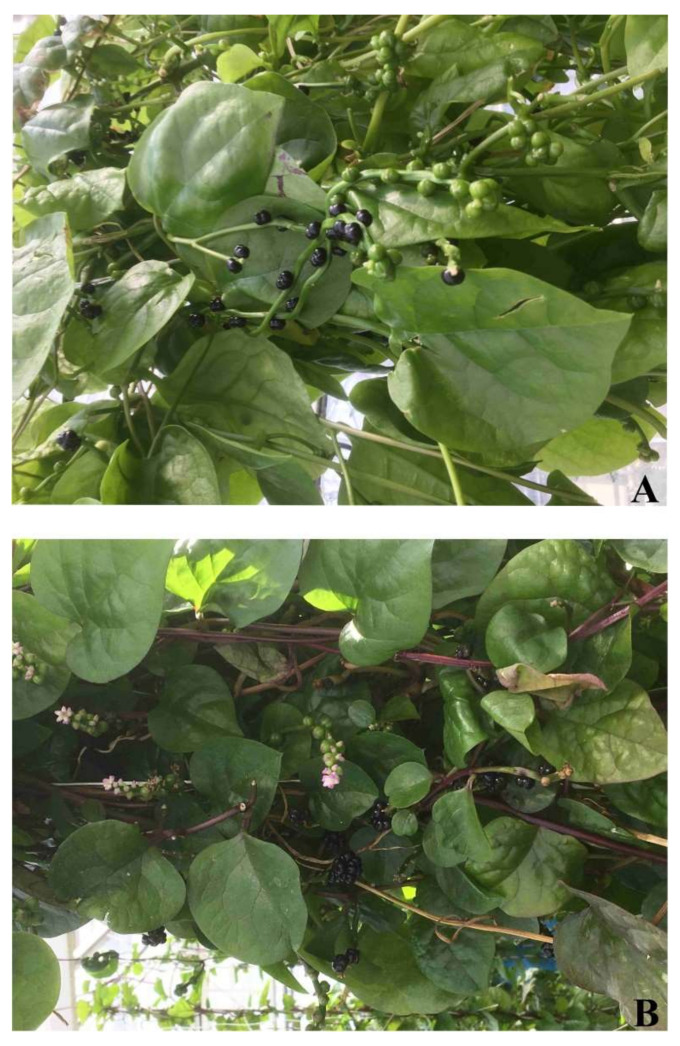
A photograph of grown *B. alba* (**A**) and *B. alba* var. ‘Rubra’ (**B**) plants.

The popularization of innovative nutraceuticals and functional foods has triggered research and exploration of alternative nutrients, including undoubtedly *Basella alba* L. and its variety *Basella alba* L. var. ‘Rubra’ (Figure 1), frequently known as Malabar spinach. These plants most widely cultivated in Asia belong to the *Basellaceae* family and are characterized by branched, climbing stems with alternating succulent and mucilaginous leaves. In summer, they abound with dark violet-blue, small stone fruits [1]. Traditional medicine, especially in India and China, uses different parts of said plants to treat many diseases [2]. In addition to the content of vitamins and minerals, the extracts of these plants can be attributed to antimicrobial, [3] anti-inflammatory and antidepressant properties [2]. Valuable sources of pro-health substances are both the stems and leaves of Malabar spinach [4], but also extracts from its fruits, which have been proven, among others, to show cytotoxic properties against human cervical cancer cells [5]. Malabar spinach fruits contain carbohydrates, proteins, lipids, niacin, ascorbic acid, tocopherols, as well as pigments—betalains, of which gomphrenin I (betanidin 6-*O*-*β*-D-glucoside) and its isoform dominate [6,7,8].

**Figure 2:**
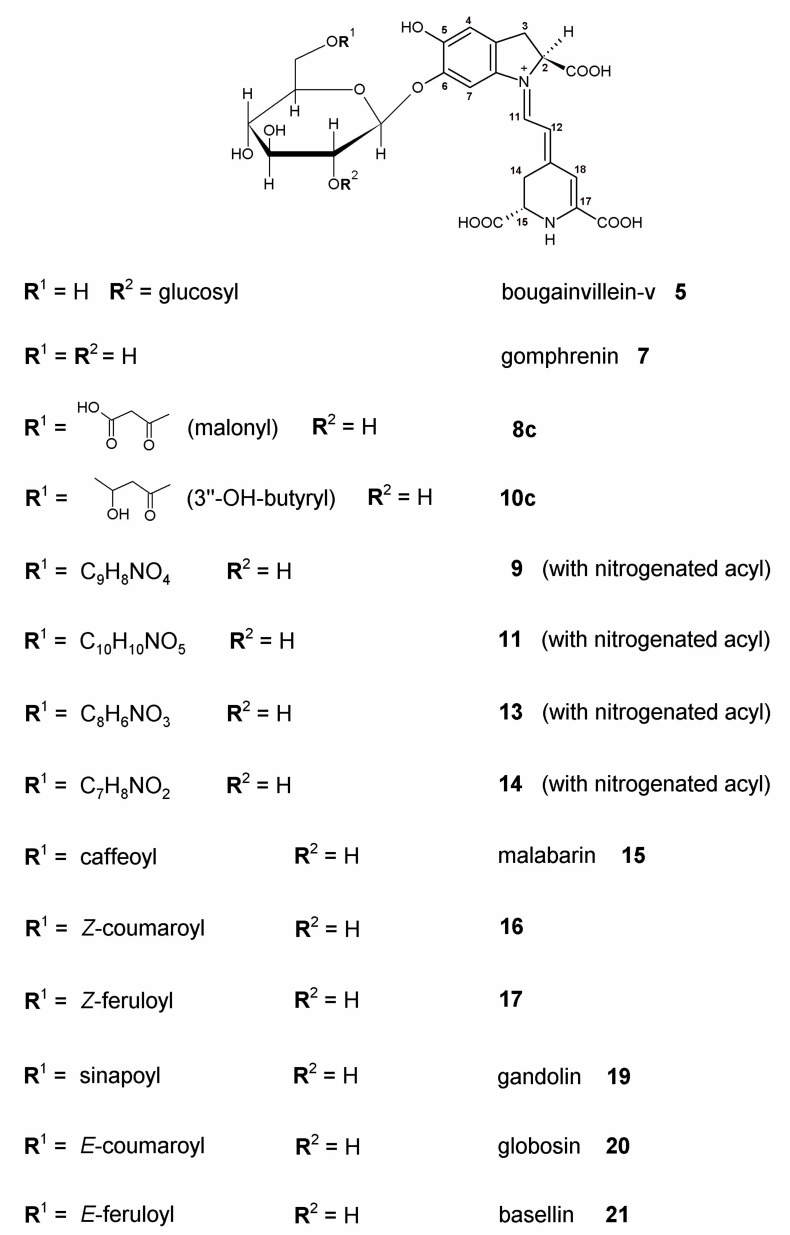
Chemical structures of the detected gomphrenin-based pigments *in* *B. alba* and *B. alba* var. ‘Rubra’ fruit juices with novel acylated betacyanins.

Betalains are a group of water-soluble colored compounds containing nitrogen in their structure (Figure 2) [7,9]. They occur in most plants of the order *Caryophyllales* [10], and they were also found in some species of fungi of the genera *Amanita* and *Hygrocybe* [11,12]. These compounds consist of betalamic acid as the chromophore core that condenses with *cyclo*-DOPA or amino acids/amines to form red-violet betacyanins and yellow-orange betaxanthins, respectively. Gomphrenins, classified as betacyanins [7], are characterized by a hydroxyl group attached to the C-5 carbon and a glucose linked at the C-6 position [13]. Betacyanins found in plant extracts are usually accompanied by their respective isoforms (isobetacyanins). The reason for isomerization may be factors induced by the environment, e.g., postharvest change of reaction pH, as well as thermal treatment.

Different forms of betacyanins behave differently depending on the conditions, e.g., thermal treatment may increase the amount of one isoform, thereby reducing the amount of the other [14].

The pigment profile of fruit extracts is influenced by the extraction method and depends on the subsequent purification method of the betacyanin fractions. Effective in terms of efficiency and economy is, among others, the purification on ion exchange beds [15,16]. An interesting literature report is the purification of extracts by pre-fermentation [17].

Betalain pigments are strong antioxidants [12] which is valuable, e.g., due to the known participation in the reduction of reactive oxygen (ROS) and nitrogen (RNS) species in the development of cancer and other diseases, such as atherosclerosis. Compounds belonging to the group of antioxidants have also been found to inhibit or delay the development of certain neoplasms [18]. The literature provides reports on the ability of betalains to inhibit the proliferation of melanoma cancer cells as well as inhibit the development of prostate and breast cancer. Moreover, no significant negative impact of these compounds on the human body has been found [19]. Based on the above facts, betalains, including betacyanins, are natural chemopreventive compounds, although they are not yet fully studied [8].

In addition to the aforementioned pro-health potential, betacyanins can be used in many fields. Due to the high coefficient of molar extinction, the dyeing capacity of betalains is competitive with that of synthetic dyes [20]. Unlike the acid-stable anthocyanins popular in coloring food, betalains maintain their color fairly well over a broader pH range (3–7). However, the most optimal condition for them is an environment with a pH in the range of 4–6 and the stability increases under anaerobic conditions [21]. Changing to a lower pH, the absorption maximum is shifted towards shorter wavelengths [7]. The stability of betalains is closely related to their chemical structure, and the improvement in stability as a result of glycosylation and acylation with hydroxycinnamic acids was indicated [22].

Raw materials rich in betalains can be used in the production of functional food where betalains not only have a color function but also increase the nutritional value. Preliminary results show the possibility of their use while maintaining stability and health-promoting properties in the dyeing of banana and lemon juices [23] or ice creams [24]. Also of interest is the confirmed encapsulation of betalain extracts from *B.* var. ‘Rubra’ juices in the form of gummy candy [25].

The not fully explored properties and unexplored possibilities of using plants rich in betalains [26,27,28], as well as the presence of unknown compounds in the profiles of extracts from individual plants, make this topic very demanding for the development of science and trends in functional food and nutraceuticals.

This contribution reports on detailed profiles of betacyanins in fruits of *B. alba* and *B.* var. ‘Rubra’ which were completely overlooked in an increasing number of reports but represent a significant fraction of the novel pigments in the fruits.

## 2. Results and Discussion

Recent reports on *B. alba* betacyanins [2,3,4,5,6,17,23,24,25] focused on the main pigment, gomphrenin, only scarcely mentioning some other acylated gomphrenins which were structurally identified two decades ago as the principal acylated pigments in purple *Gomphrena globosa* L. [7,29]. In this study, our detailed studies evidently detected new betacyanin pigments (Table 1, Figure 2) and also revealed completely novel structures with unique acylation patterns (Table 2 and Table 3) as well as compared quantitatively the two plant varieties (Section 2.7) [30]. Determination of unequivocal elemental compositions of unknown genuine pigments was possible using the HRMS coupled to the HPLC separation system. 

The ‘soft’ fragmentation experiments at an applied energy of 20 eV in the quadrupole collisional stage before the Orbitrap HRMS detection gave especially valuable information concerning the main part structures of the analyzed compounds.

**Figure 3:**
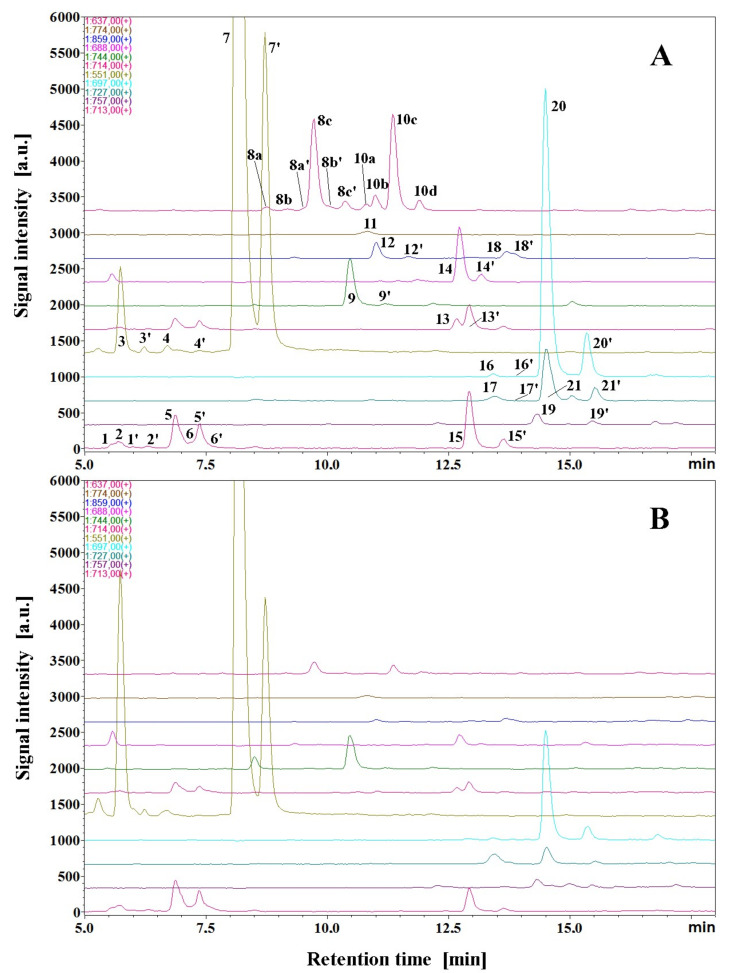
Betacyanin fingerprints in *B. alba* (**A**) and *B. alba* var. ‘Rubra’ (**B**) fruits in the form of high-performance liquid chromatograms for selected ions obtained in the LRMS LC-DAD-MS system.

The resulting betacyanin fingerprints in *B. alba* and var. ‘Rubra’ fruits in the form of chromatograms for selected ion monitoring obtained in the LC-DAD-MS system are presented in Figure 3A,B, respectively. The presence of unique betacyanins with acylating moieties containing nitrogen in their structures is confirmed in the samples based on the following analytical HRMS results. The complete ^1^H, ^13^C and 2-D NMR data were obtained for the principal acylated betacyanins isolated from *B. alba* fruits (**15**, **19**, **20** and **21**) for the first time (Section 2.6.).

### 2.1. Non-Acylated Polar Betacyanins Identified in the Fruits of B. alba and var. ‘Rubra’

In the group of polar betacyanins identified in the fruits of *B. alba* and var. ‘Rubra’ by LC-DAD-MS and co-elution experiments with the known references [7,26,28,29,30,31,32], except for the known mono- and bi-glucosylated betacyanins **1**, **4, 5** and **7** as well as their isoforms **1**’, **4**’, **5**’ and **7**’ (Table 1), novel pigments **2**/**2**’, **3**/**3**’ and **6**/**6**’ were detected, which were not co-eluted with the reference betacyanins.

Betacyanins **2** and **6** showed protonated molecular ions at *m*/*z* 713 as well as their daughter ion fragments at *m*/*z* 551 and 389, respectively, in the positive ion mode LC-MS/MS (Table 1). The molecular mass and the fragmentation pattern suggested a presence of a dihexosyl (713 − 389 = 2 × 162) of betanidin. The observed low absorption maxima λ_max_ 536–537 nm suggested the 5-*O*-substitution pattern with a sugar system in betanidin, similarly to melocactin [7,28]. During the high-resolution mass spectrometric experiments on compounds **2** and **6** in the Orbitrap system, the molecular masses were obtained at *m*/*z* 713.2030 and 713.2033, respectively (C_30_H_37_N_2_O_18_, calculated *m*/*z* 713.2036), which together with the detected fragmentation ions (Table 2) confirmed the elemental composition of **2** and **6**.

Unexpectedly, compound **3** with [M+H]^+^ ions at *m*/*z* 551 appeared as a hexosyl (551 − 389 = 162) of betanidin during the low-resolution mass spectrometric analysis, which together with the observed higher absorption maximum λ_max_ 539 nm (Table 1) tentatively suggested a presence of a betanidin 6-*O*-substitution system. The 2 nm difference in λ_max_ is always observed between betanins and gomphrenins acylated with aliphatic acids [7,28,31]. The obtained protonated molecular mass for **3** during the HRMS experiments corresponding to the ion at *m*/*z* 551.1505 (C_24_H_27_N_2_O_13_, calculated *m*/*z* 551.1508) and for its fragment ion (Table 2) at *m*/*z* 389.0973 (C_18_H_17_N_2_O_8_, calculated *m*/*z* 389.0979) confirmed a presence of a novel isomeric betacyanin to the well-known betanin **4** and gomphrenin **7**.

### 2.2. Malonylated Betanidin 6-O-β-Glusosides and Their Acyl Migration Derivatives

Further inspection of the chromatograms of the fruit extracts revealed two main peak groups with protonated molecular ions at *m*/*z* 637, **8a**–**8c**/**8a**’–**8c**’ and **10a**–**10d**, apparently isomeric to phyllocactins [7,26,33]. The HPLC co-elution experiments with the authentic standards of phyllocactin/isophyllocactin from *Hylocereus ocamponis* fruits [26] excluded the presence of phyllocactin/isophyllocactin in the samples. Higher retention times of **8a**–**8c**/**8a**’–**8c**’ and **10a**–**10d** than those of phyllocactins [26] suggested possible malonylation of gomphrenin/isogomphrenin at least in one of the detected groups, which is the first example of malonylation of gomphrenins.

This finding was supported by fragmentation experiments of **8a**–**8c**/**8a**’–**8c**’ of the protonated molecular ions (Table 1) and detection of the fragments at *m*/*z* 619 (- H_2_O), 593 (- CO_2_), 551 and 389, suggesting the presence of malonylated (637 − 551 = 86) hexose (551 − 389 = 162). However, similar results were also obtained for the pigments **10a**–**10d**.

The decisive data (Table 2) were obtained by the high-resolution experiments in the Orbitrap system and confirmed that the structures of **8a**–**8c**/**8a**’–**8c**’ fit to the malonylated betacyanins (obtained mass for the most abounding isomer **8c** at *m*/*z* 637.1513 (C_27_H_29_N_2_O_16_, calculated mass: 637.1512)), at the same time excluding the presence of malonylated derivatives in **10a**–**10d** (discussed in the next section).

The position of 6-*O*-glucosylation in **8a**–**8c**/**8a**’–**8c**’ might be suggested by a bathochromic shift of their obtained absorption maxima λ_max_ (537 nm), similar to the shift from 535 nm for betanin to 537 nm for gomphrenin in the applied chromatographic eluent [34]. This is the always-observed difference between betanins and gomphrenins acylated with aliphatic acids [7,28,31,34].

The presence of the characteristic pigment pattern observed in **8a**–**8c**/**8a**’–**8c**’ additionally indicates a possibility of the presence of acyl-migrated stereoisomers (the minor 3’-*O*- and 4’-*O*-malonylated acyl-migration products **8a**–**8b**/**8a**’–**8b**’) and the main 6’-*O*-malonylated forms **8c**/**8c**’**.** Their presence is evidently predicted based on previous studies performed on betanin-like malonylated betacyanins [7,26,33,35]. Both the 3’-*O*- and 4’-*O*-malonylated gomphrenin pairs **8a**–**8b**/**8a**’–**8b**’ are most presumably eluted before the main peaks of the corresponding 6’-*O*-malonylated forms **8c**/**8c**’ (Figure 3).

### 2.3. 3-Hydroxy-Butyrylated Betanidin 6-O-β-Glusosides

Pigments **10a**–**10d**, which were apparently isomeric to phyllocactins, were characterized with protonated molecular ions at *m*/*z* 637 and absorption maxima λ_max_ 537–538 nm in the applied chromatographic eluent. Submitted to the LRMS analyses, formed fragmentation ions at *m*/*z* 593, 551 and 389 were similar to those obtained for the betacyanins **8a**–**8c**/**8a**’–**8c**’. Subsequent HRMS experiments on compounds **10a**–**10d** in the Orbitrap system excluded the presence of typical malonylated structures, instead, the acyl identity was readily proposed as 3’’-hydroxy-butyryl for all the isomers **10a**–**10d** yielding, e.g., *m*/*z* 637.1875 for the most prominent pigment **10c** (C_28_H_33_N_2_O_15_, calculated *m*/*z* 637.1876), which suggested a presence of a 3’’-hydroxy-butyrylated hexosyl of betanidin/isobetanidin (Table 3). Taking the above data into consideration, some of the pigments **10a**-**10d** can be tentatively identified as 3’’-hydroxy-butyrylated gomphrenin/isogomphrenin (presumably the more abundant pair **10c**/**10d** (Section 2.7). The lack of a carboxylic moiety in the 3’’-hydroxy-butyryl substituent prevents the occurrence of the phenomenon of acyl migration in **10a**–**10d**, therefore, one of the pairs might be betanin derivatives.

The 3’’-hydroxy-butyryl substitution had been already tentatively suggested for the structures of the phyllocactin/isophyllocactin additional isomers detected in *H. polyrhizus* fruits [36], but further extensive studies proved that these isomers were the acyl migration products and no 3’’-hydroxy-butyryl substitution could be considered at all [26,33]. Unfortunately, other recent reports (data not shown) cited this tentative assumption for other genera of *H. polyrhizus* without any proof and without taking the acyl migration into consideration. Now, according to our best knowledge, this study reports the first cases of tentatively identified 3’’-hydroxy-butyrylated betacyanins **10a**–**10d**.

### 2.4. Hydroxycinnamic Acid Conjugates of Gomphrenin

A consequent search for a group of novel hydroxycinnamoylated gomphrenins, except previously structurally elucidated 4-coumaroylated gomphrenin (gomphrenin II, globosin) **20** and feruloylated gomphrenin (gomphrenin III, basellin) **21** as well as tentatively identified sinapoylated gomphrenin (gomphrenin IV, gandolin) **19** [7,29,31], resulted in the identification of caffeoylated gomphrenin **15** as well as isomeric structures of coumaroylated dihexosylated betanidin **12** and **18** in this study.

The observed fragmentation pathway for the protonated molecular ion of **15** afforded fragments at *m*/*z* 551 and 389 (Table 2), indicating detachment of a caffeoyl moiety (713 − 162 = 551 Da) presumably at the glucosyl residue of gomphrenin (551 − 389 = 162). A high retention time of **15** supported the presence of acylated betacyanin similar to the other acylated pigments **16**–**21**.

The high-resolution mass spectrometric determination of the molecular mass for **15** by obtaining *m*/*z* 713.1820 (C_33_H_33_N_2_O_16_, calculated *m*/*z* 713.1825) as well as for its decarboxylated derivatives (Table 2) readily confirmed the substitution with the acyl moiety instead of another hexosyl in **15**, thus, proposing its tentative identity as 6’*O*-*E*-caffeoyl-gomphrenin and a trivial name of “malabarin” of this endogenously present pigment in *B. alba* (Malabar spinach). Our NMR analysis of **15** finally confirmed this structure (Section 2.6).

Initial analyses of the structures of **12** and **18** brought the protonated molecular ions at *m*/*z* 859 and their fragmentation resulting in fragments at *m*/*z* 697, 551 and 389 (Table 2), indicating a detachment of a hexosyl (859 − 162 = 697 Da) and coumaroyl moiety (697 − 162 = 551 Da) from the first hexosyl attached to betanidin (551 − 389 = 162). Because these compounds might be gomphrenin derivatives, subsequent co-elution tests with *B. glabra* known pigments were performed and excluded the presence of betanidin 6-*O-*(6’-*O*-*E*-4-coumaroyl)*-β*-sophoroside and betanidin 6-*O-*(6’’-*O*-*E*-4-coumaroyl)*- β*-sophoroside.

The HRMS experiments on **12**/**18** confirmed the proposed acylation of a dihexosyl-betanidin system with a coumaroyl moiety based on obtained protonated molecular ions at *m*/*z* 859.2398 and 859.2407, respectively (C_39_H_43_N_2_O_20_, calculated mass: 859.2404), as well as on their fragmentation ions (Table 2). Thus, the tentative structures of **12** and **18** are proposed as isomers of (hexosyl)-(coumaroyl-hexosyl)-betanidin.

Previously detected sinapoyled gomphrenin **19** in purple inflorescences of *G. globosa* [34], in this study, was first ascertained in the *B. alba* and var. ‘Rubra’ fruits by co-elution experiments. Its molecular formula was obtained by HRMS analysis (Table 2), yielding the protonated molecular ion at *m*/*z* 757.2083 (C_35_H_37_N_2_O_17_, calculated mass: 757.2087) and its fragmentation ions at 713.2178 (- CO_2_), 669.2291 (- 2CO_2_), 551.1502 (- sinapoyl) and 389.0973 (- sinapoyl-glucosyl). Subsequently, the NMR analysis finally confirmed the structure of **19** (Section 2.6).

Similarly, the cis-isomers of hydroxycinnamic acid conjugates of gomphrenin **16**/**17** as well as their isoforms **16**’/**17**’ were detected in the studied fruits, which was corroborated by the co-elution experiments with references obtained from *G. globosa* inflorescences and HRMS measurements (Table 2) [34].

### 2.5. Novel Natural Betacyanins Acylated with Nitrogenous Substituents

The most engaging was the detection of unusual betacyanins **9**, **11**, **13** and **14** as well as their isoforms **9**’**, 11**’**, 13**’ and **14**’ acylated with acids containing nitrogen in their structures. From this group of pigments contributing to a level of 4.4% of the total pigment content in fruits of *B. alba*, the most abundant were **9** and **14** (Section 2.7).

Chromatographic LC-DAD-MS/MS analyses of **9** with LRMS detection revealed its absorption maximum λ_max_ 543 nm typical for hydroxycinnamic acid conjugates of gomphrenin or betanin, however, protonated molecular ions detected at *m*/*z* 744 indicated acylation with a nitrogenous moiety. After fragmentation experiments, a more rich fragmentation pattern was obtained for **9** with ions at *m*/*z* 700 (- CO_2_); 656 (- 2CO_2_); 612 (- 3CO_2_); 568 (- 4CO_2_); 551 (- acyl); and 389 (- acyl-glc), suggesting the presence of a betacyanin with four carboxylic groups. Therefore, it can be assumed that a nitrogenous acyl group containing one carboxyl is attached to gomphrenin in **9**.

Subsequent HRMS experiments on compound **9** in the Orbitrap system confirmed these assumptions, yielding protonated molecular ions at *m*/*z* 744.1878 (C_33_H_34_N_3_O_17_, calculated *m*/*z* 744.1883), which readily disclosed the molecular formula of the acyl moiety (C_9_H_8_NO_4_). This was also confirmed by the detection of the other fragments in the HRMS mode (Table 3). Interestingly, this result would fit with the presence of betalamic acid as acylating agent; however, the lack of an additional absorption maximum around λ_max_ 422 nm, typical for this pigment, as well as the not increased reactivity of **9,** which would be ascribed to the aldehyde functional group, suggests that this cannot be the case. Instead, rather another form of acyl related to betalamic acid should be considered.

Similar results were observed for the pigment **14** with lower absorption maximum λ_max_ 538 nm and protonated molecular ions detected at *m*/*z* 688. The fragmentation pattern obtained for **14** was less abundant and accounted for ions at *m*/*z* 644 (-CO_2_); 600 (-2CO_2_); 551 (-acyl); and 389 (-acyl-glc), thus, indicating a presence of a betacyanin with a nitrogenous acyl group but not containing any carboxyl. In the HRMS experiments, a protonated molecular ion at *m*/*z* 688.1983 was reported (C_31_H_34_N_3_O_15_, calculated *m*/*z* 688.1984), which revealed the chemical formula of the acyl moiety (C_7_H_8_NO_2_). The other fragments in the HRMS mode were also detected and unequivocally confirmed the molecular formula of **14** (Table 3).

Furthermore, the presence of pigments **11** and **13** was revealed in both the *B. alba* and var. ‘Rubra’ fruits, which appeared as differing from the structure of pigment **9** by a presence or absence, repectively, of a methoxy group resulting from the LC-DAD-MS/MS detection of protonated molecular ions at *m*/*z* 774 and 714, respectively, as well as the absorption maximum λ_max_ 542 nm. In spite of the small quantities of pigment **11** (Table 3), it was possible to obtain, albeit scarce, fragmentation patterns with ions observed at *m*/*z* 742 (-CH_3_OH) and 389 (-acyl-glc), confirming a presence of methoxyl in the nitrogenous acyl moiety. Unexpectedly, no typical ion at *m*/*z* 551 was detected during the fragmentation, which would confirm the glucosylated betanidin fragment. In contrast, a more abundant fragmentation profile was monitored for **13** with ions at *m*/*z* 670 (-CO_2_); 636 (-2CO_2_); 582 (-3CO_2_); 551 (-acyl); 538 (-4CO_2_); and 389 (- acyl-glc), which was similar to the profile obtained for **9**, also confirming four carboxylic groups present in a betacyanin **13**.

The experiments performed in the HRMS mode in the Orbitrap system revealed much more prolific fragmentation patterns obtained for compound **11** after fragmentation of the protonated molecular ion at *m*/*z* 774.1975 (C_34_H_36_N_3_O_18_, calculated *m*/*z* 774.1988), accounting for demethoxylation (*m*/*z* 742.1715 (C_33_H_32_N_3_O_17_, calculated *m*/*z* 742.1726)) of the [M+H]^+^ ion and further four decarboxylation steps of the demethoxylated ion (Table 3) as well as deacylation and deglucosylation as in the case of **9**. The chemical formula of the acyl moiety in **11** was established as C_10_H_10_NO_5_.

Determination of the molecular formula of [M+H]^+^ ions of compound **13** in the HRMS mode (*m*/*z* 714.1776 (C_32_H_32_N_3_O_16_, calculated *m*/*z* 714.1777)) afforded to establish the chemical formula of the acyl moiety in **13** as C_8_H_6_NO_3_. This was unequivocally confirmed by the determination of the elemental composition of the fragmentation ions (Table 3).

### 2.6. NMR Structural Elucidation of Acylated Gomphrenins

Isolated quantities of **15**, **19**, **20** and **21** enabled their first complete structural analysis by two-dimensional NMR techniques. The characteristic signals of the aglycone and glucose moieties [7,26,31,35,37,38,39,40] in the spectra of **15** (Figures S1 and S2), **19** (Figures S3 and S4), **20** (Figures S5 and S6) and **21** (Figures S7 and S8), confirmed the presence of gomphrenin-derived pigments. The three individual coupled ^1^H-spin systems of the aglycone (H-2, H-3ab; H-11, H-12; H-14ab, H-15) were assigned in ^1^H NMR as well as COSY and TOCSY spectra [7,26,31,35,37,38,39,40]. In each case, the betanidin system was readily distinguishable by the characteristic low- and high-field doublet signals for the H-11 and H-12 protons.

The spin system for H-2/H-3ab was observable in each pigment, indicating the presence of the carboxyl moiety at C-2. A significant upfield shift for the H-2 proton to 3.79–3.88 ppm (in **15**, **20** and **21**) or 4.72 (in **19**) in comparison to non-acylated gomphrenin **7** was observed, suggesting intramolecular interactions [31,40]. This phenomenon is still not explored but is additionally suggested by the observed bathochromic shift of the visible absorption band in gomphrenins in relation to betanins [7,31].

The other spin system, for H-15/H-14ab, showed easily identifiable cross-peaks in the COSY and TOCSY spectra, also resulting from the presence of the carboxyl moiety at C-15.

The three singlets corresponding to H-4, H-7 and H-18 were detected in the spectra. A broad signal for H-18 in **15**, **20** and **21** was observable by ^1^H NMR and the correlation techniques for freshly prepared D_2_O solutions of the pigments avoiding the fast deuterium exchange [40]. In the case of **19**, the acidic CD_3_OD solutions enabled observation of a stable narrow signal.

In contrast to previous reports [31,40], the presence of the characteristic interconnection system for gomphrenin could not be indicated (except of **19**) based on the shift differences between H-4 and H-7 of 0.6-0.8 ppm, presumably because the measurements were performed in D_2_O instead of acidified CD_3_OD. Therefore, the well-developed confirmation of the C-6 phenolic moiety substitution in the betanidin system was obtained by the other techniques (NOESY and HMBC). Furthermore, this shift difference was not only lower than 0.2 ppm but also varied within the group of the studied acylated betacyanins **15**, **20** and **21** (Table 4). In the case of the C-5 substitution, the expected differences of ca. 0.1 ppm or lower were observed in acidified CD_3_OD [40] but also in acidified D_2_O [37] and non-acidified D_2_O [35,37].

**Figure 4:**
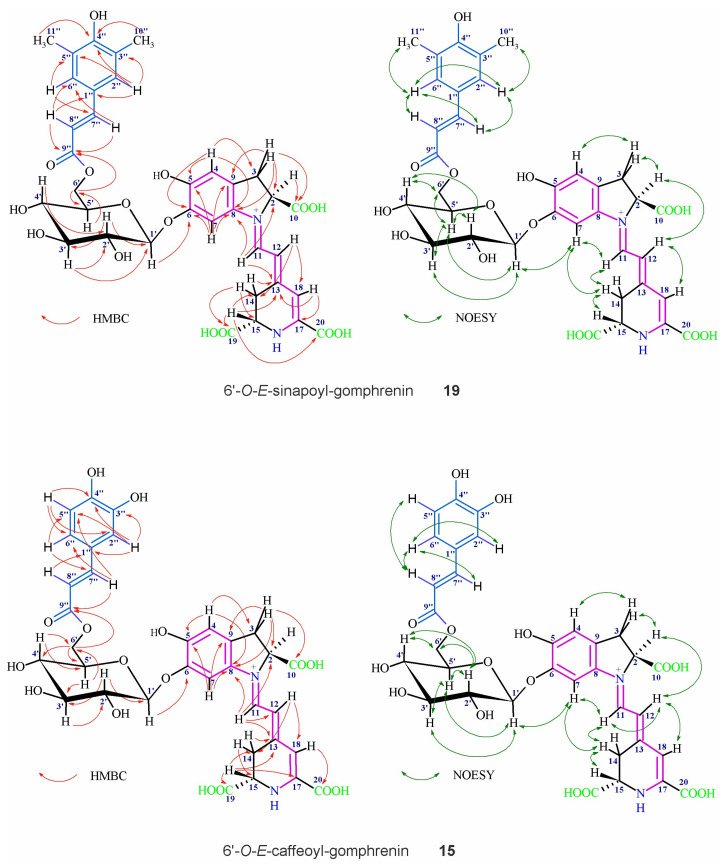
Important HMBC and NOESY NMR correlations indicating the structures of the chromophoric systems and the positions of the glycosidic bonds as well as the acyl moieties in the novel betacyanins: 6’*O*-*E*-caffeoyl-gomphrenin (malabarin) **15** and 6’*O*-*E*-sinapoyl-gomphrenin (gandolin) **19**.

The dihydroindolic system was assigned by HSQC correlations of H-2, H-3ab, H-4 and H-7 with their respective carbons. The correlations of C-5 to H-4/H-7, C-6 to H-4, C-8 to H-3ab, H-4, H-7 and H-11, C-9 to H-7/H-3ab and C-10 to H-3ab (the dihydroindolic system) as well as C-12 to H-11, C-13 to H-11, H-15 and H-14ab, C-14 to H-12 and H-15, C-18 to H-12, C-19 to H-15, and C-20 to H-11 (the dihydropyridinic system) were determined by HMBC in D_2_O but also in H_2_O/D_2_O (90/10, *v*/*v*) (when necessary for obtaining the signals of exchangeable proton H-18) (Figure 4, Table 4).

Additional data observed for the chromophoric systems in the NOESY spectra confirmed the key correlations (Figure 4) of the proton H-15 with H-14a/b as well as between H-7, H-11 and H-14a/b. Together with correlations of H-12 with H-2 and H-18, these data confirmed the principal *E*-configuration for C(12)=C(13) and s-*trans* conformation for the dienyl moiety N(1)=C(11)-C(12)=C(13) in the most abundant stereoisomer [40].

The presence of the *Z*-isomers (data not shown) was also acknowledged for the acylated gomphrenins **15**, **19**, **20** and **21** [31,40]. The correlations between the *E*- and *Z*-protons being in equilibrium (at the signal ratio ca. 90:10) were noticeable in the TOCSY and NOESY spectra. The signals of the *Z*-protons were detected for the betanidin system (H-2, H-3ab, H-4, H-7, H-11, H-12, H-14ab and H-15) as well as for the caffeoyl moiety (H-2″, H-5″, H-6″, H-7″ and H-8’’). Due to low signal intensities, the expected cross-peaks for the *Z*-protons of H-12 and H-14ab confirming the *Z*-configuration for C(12)=C(13) were not observable in the NOESY spectra. Such correlations were observed for more abundant (35%) *Z*-isomers in the other betalainic group, betaxanthins [41].

The other ^13^C chemical shifts for carbons directly bound to protons were assigned by HSQC correlations. The presence of the anomeric proton H-1′ indicating the sugar unit by its characteristic downfield shift was readily observed. The HMBC, COSY and TOCSY correlations clearly ascertained the glucosyl ring systems (Figure 4, Table 4) [7,26,31,35,37,38,39,40]. The position of the glycosidic bond at the phenolic carbon C-6 was readily confirmed by the HMBC correlation with the anomeric proton H-1’. The *β*-linkage between the aglycone and glucopyranosyl moiety was denoted by the three-bond vicinal proton coupling constant ^3^*J*_1’-2’_ ~6–7 Hz after re-registration of the ^1^H spectra in other CD_3_OD/d-TFA solutions [37,40].

Definitive evidence of the acyl moiety position was provided by the downfield chemical shift of H-6’a/b protons in the glucosylic ring. Further confirmation of this linkage position was obtained by the HMBC correlations (Figure 4) of C-9’’ to H-6’a and H-6’b.

The hydroxycinnamic acyl moieties were readily detected by their aromatic and olefinic protons (*J* = 16 Hz) and were differentiated by the presence or absence of the hydroxyl and methoxyl moieties at carbons C-3’’ and C-5’’ (Figure 4, Table 4).

Above analyses completed the structural identification of the novel betacyanins: 6’*O*-*E*-caffeoyl-gomphrenin (proposed trivial name: malabarin) **15** and 6’*O*-*E*-sinapoyl-gomphrenin (gandolin) **19** as well as further two-dimensional characterization of 6’-*O*-*E*-4-coumaroyl-gomphrenin (globosin) **20** and 6’-*O*-*E*-feruloyl-gomphrenin (basellin) **21**.

### 2.7. Quantification of Betacyanins in the Fruits of B. alba and B. alba var. ‘Rubra’

For *B. alba* var. ‘Rubra’, a much higher total concentration of betacyanins expressed in betanin equivalents (Table 5) was obtained in the mature fruits (86.6 mg/100 g) than for *B. alba* (42.0 mg/100 g). This is roughly in accordance with previous reports on single varieties of the species [5,42]. The distribution of the pigments is also much different in both the fruit types. In *B. alba*, the fraction of acylated betacyanins is much higher (38.6%) than in var. ‘Rubra’ (19.4%). Similarly, the percentage of the novel nitrogenous betacyanins in *B. alba* (4.4%) is twice as much as the fraction in var. ‘Rubra’ fruits (2.2%).

The most abundant acylated pigment, (6’*O*-*E*-4-coumaroyl)-gomphrenin (globosin, former gomphrenin II) **20**, was reported at a fraction of 12.9% in *B. alba* and 6.6% in var. ‘Rubra’ fruits. The other relatively higher concentrations in *B. alba* fruits were reported for the novel acylated pigments, 6′-*O*-malonyl-gomphrenin **8c** (3.9%) and one of the isomers of 3″-hydroxy-butyryl-gomphrenin **10c** (4.4%).

From the polar pigments, gomphrenin **7** contributed to the total betacyanin content at 43.9% and 39.7% in var. ‘Rubra’ and *B. alba*, respectively. The portion of isogomphrenin **7**’ was reported at 13.2% and 13.7% in var. ‘Rubra’ and *B. alba*, respectively. Unexpectedly, the contribution of betanin **4** to the betacyanin total content (0.44% and 0.37% in var. ‘Rubra’ and *B. alba*, respectively) was much smaller than the fraction of the novel isomeric pigment, (hexosyl)-betanidin **3**, which accounted for 17.5% and 3.7% in var. ‘Rubra’ and *B. alba*, respectively.

## 3. Materials and Methods

### 3.1. Reagents

All reagents were used as received. Formic acid, LC-MS grade methanol, and water were obtained from Sigma Chemical Co. (St. Louis, MO, USA). The deionized water used throughout the experiments was purified through a Purix water purification system with a resistivity of 18.0 mΩ cm^−1^ at 295 K.

### 3.2. Plant Material

The seeds of *Basella alba* L. and *Basella alba* L. var. ‘Rubra’ obtained from the Botanical Garden of Jagiellonian University Institute of Botany (Cracow, Poland) were grown in a greenhouse of the University of Agriculture in Cracow (Faculty of Biotechnology and Horticulture). Sowing of the seeds was performed in a 3:1 ratio of soil and coconut pith mass and watered daily. The seedlings were transplanted to fertile soil with plenty of organic matter and a pH of 6.5–6.8. The plants were designed to support the climbing of the vines and were fast growing; therefore, they were trellised so that they reached up to 3 m in height. The plants were kept at consistent moisture and temperature to keep flowering and fruiting.

### 3.3. Betacyanin Reference Material

*Gomphrena globosa* L. inflorescences, *Bougainvillea glabra* Choisy bracts, *Mammillaria coronata* (Scheidweiler) fruits, *Schlumbergera* x *buckleyi* (T. Moore) Tjaden sunlight-stressed leaves and *Portulaca oleracea* stems were obtained from the Botanical Garden of Jagiellonian University Institute of Botany (Cracow, Poland) [7]. *Hylocereus ocamponis* fruit peel extract was obtained from a previous study [7,26].

### 3.4. Preparation of Juice from B. alba and B. var. ‘Rubra’ Fruits

The fruits collected in the greenhouse (30 g for each variety) were squeezed and obtained liquid was centrifuged followed by filtering through a 0.2 mm i.d. pore size filter and then underwent threefold dilution with water before immediate chromatographic analysis of the pigment profiles or storage at −20 °C before the subsequent experiments.

### 3.5. Fast Betacyanin Screening in the Fruit Juice Samples

Betacyanin samples from the prepared fresh fruit juice of *B. alba* and var. ‘Rubra’ were immediately submitted to spectrophotometric as well as LC-MS analysis without any purification. For the pigment profile representation, a method of internal normalization of the chromatographic peaks derived from the MS signals was applied. For the measurement of the total concentration of the pigments, the extracts were analyzed by an Infinite 200 microplate reader (Tecan Austria GmbH, Grödig/Salzburg, Austria). The total concentration was expressed as mg betanin equivalents/100 g of fresh fruits. Quantification of betacyanins was evaluated taking a molar extinction coefficient of ε = 65,000 M^−1^ cm^−1^ at 536 nm for betanin in spectrophotometric calculations [30]. Three samples per species were analyzed according to this procedure.

### 3.6. Pigment Purification for LC-MS Experiments

For the further LC-MS analyses with detection by low- and high-resolution mass spectrometry, purification of *B. alba* extracts was performed to obtain preconcentrated samples. The pigment extracts were chromatographically purified by flash chromatography using a Shimadzu LC-20AD preparative chromatographic system (Kyoto, Japan) equipped with LC-20AP pumps, SPD-20AV UV−Vis detector, and LabSolutions 5.51 operating software. The separation was performed on Bionacom cartridges (Agela Technologies, Newark, DE) filled with non-endcapped silica C18 sorbent (porosity 60 Å and particle size 40–60 μm) [28]. After rinsing with water, the betacyanin fraction was eluted with 50% aqueous methanol acidified with 5% formic acid (v/v). The eluates were pooled and concentrated using a rotary evaporator under reduced pressure at 25 °C and freeze-dried. A similar purification procedure was performed for betacyanins from the reference material samples.

### 3.7. Preparation of Isolated Betacyanins from the Purified B. alba Extract

For the NMR study, betacyanins **15**, **19**, **20** and **21** were isolated from *B. alba* extract by chromatographic steps. The extract was initially purified by open column chromatography on a column (40 mm i.d. × 50 mm height) filled with Sepra™ ZT-SAX 30 μm Polymer, 85-Å (Phenomenex, Torrance, CA, USA). After application of the extract to the top of the column and rinsing the column with water, the betacyanin fraction was eluted with 50% aqueous methanol acidified with 5% formic acid (*v*/*v*). The eluates were pooled and concentrated using a rotary evaporator under reduced pressure at 25 °C before purification by flash chromatography on non-endcapped silica C18 sorbent (as described in Section 3.6) in a column of 40 mm i.d. × 140 mm height.

The concentrated eluates from the silica C18 sorbent were pooled and the pigments were separated using the Shimadzu LC-20AD system on an HPLC semipreparative column Synergi Hydro-RP 250 mm × 30 mm i.d., 10 μm (Phenomenex) with a 20 mm × 25 mm i.d. guard column of the same material (Phenomenex). A typical gradient system consisting of 1% aqueous formic acid (solvent A) and acetone (solvent B) was used as follows: 0 min, 15% B; increasing to 10 min, 17% B; increasing to 20 min, 20% B; increasing to 30 min, 22% B; increasing to 40 min; 80% B. The injection volume was 20 mL with a flow rate of 30 mL/min. Detection was performed using a UV/Vis detector at 538 and 480 nm, at a column temperature of 22 °C. The eluates were pooled and concentrated under reduced pressure at 25 °C and finally freeze-dried. All the solutions were concentrated in rotary evaporators at 25 °C under reduced pressure to remove the organic solvent and stored at −20 °C for further studies.

### 3.8. Chromatographic Analysis with Detection by a Low-Resolution Mass Spectrometric System (LC-DAD-ESI-MS/MS)

For the chromatographic and mass spectrometric analyses, an LCMS-8030 mass spectrometric system (Shimadzu, Kyoto, Japan) coupled to LC-20ADXR HPLC pumps, an injector model SIL-20ACXR, and a PDA detector (photo diode array) model SPD-M20A, all controlled with LabSolutions software version 5.60 SP1 (Shimadzu, Japan), was used. The samples were eluted through a 150 mm × 4.6 mm i.d., 5.0 μm, Kinetex C18 chromatographic column preceded by a guard column of the same material (Phenomenex, Torrance, CA, USA). The injection volume was 20 μL, and the flow rate was 0.5 mL/min. The column was thermostated at 40 °C. The separation of the analytes was performed with a binary gradient elution. The mobile phases were: A—2% formic acid in water and B—methanol. The gradient profile was: (t (min), % B), (0, 10), (12, 40), (15, 80), (19, 80). The full range PDA signal was recorded, and chromatograms at 538, 505, 490 and 440 nm were individually displayed. Positive ion electrospray mass spectra were recorded on the LC-MS system, which was controlled with LabSolutions software. The ionization electrospray source operated in positive mode (ESI+), at an electrospray voltage of 4.5 kV and capillary temperature at 250 °C, using N_2_ as a gas for the spray, recording total ion chromatograms, mass spectra and ion chromatograms in selected ion monitoring mode (SIM) as well as the fragmentation spectra. Argon was used as the collision gas for the collision-induced dissociation (CID) experiments. The relative collision energies for MS/MS analyses were set at −35 V in an arbitrary scale.

### 3.9. Chromatographic Analysis with Detection by a High-Resolution Mass Spectrometric System (LC-Q-Orbitrap-MS)

All high-resolution mass spectra were analyzed using Q Exactive Plus hybrid OrbiTrap quadrupole mass spectrometer (Thermo Fisher Scientific, Brema, Germany) coupled to an HPLC Dionex UltiMate 3000 chromatographic separation system. The chromatographic conditions were the same as for the LRMS system.

The conditions for positive thermally focused/heated electrospray (HESI) were as follows: capillary voltage, 3.5 kV; capillary temperature, 250 °C; sheath gas, auxiliary gas and sweep gas flow rate were set at 50, 15 and 3 arbitrary units, respectively; probe heater temperature, 350 °C; S-lens RF level, 55%.

The detection of target betacyanins selected in the LRMS system was conducted in the full scan positive polarity mode. The MS data were acquired in the *m*/*z* 400−1000 range with a resolution (full width at half-maximum, FWHM, at *m*/*z* 200) of 70,000. The automatic gain control (AGC) target value was 200 000 in the full-scan mode. The maximum isolation time was set to auto mode.

Selected precursor ions were fragmented in the higher-energy collisional activated dissociation cell and the fragment (MS2) ions were analyzed in the Orbitrap analyzer. For the MS2 experiments, the fragment ions of selected target betacyanins were collected in the high-energy collision dissociation (HCD) mode at collision energies of 20 and 40 eV. The automatic gain control (AGC) target value and the resolution were 50,000 and 35,000, respectively. The *m*/*z* range was 70−900 and the maximum isolation time was set to auto mode. The number of microscans per MS/MS scan was set to 1. The LC-HRMS data acquisition and analysis were performed by using the software Chromeleon 7.2.10 and Xcalibur 4.3 (Thermo Fisher Scientific).

### 3.10. NMR Experiments

The NMR data were acquired on a Bruker Avance III 700 spectrometer (Bruker Corp., Billerica, MA, USA) using a QCI CryoProbe at 295 K in non-acidified D_2_O (**15**, **20** and **21**) and CD_3_OD acidified by d-trifluoroacetic acid (**19**).

All 1D (^1^H, ^13^C) and 2D NMR (COSY, HSQC, HMBC, TOCSY and NOESY (gradient enhanced)) measurements were performed using standard pulse sequences and acquisition parameters [7,26]. The residual water peak for experiments carried out in D_2_O was suppressed using the low-power presaturation. Chemical shifts were referred to internal 3-(trimethylsilyl)-2,2,3,3-tetradeuteropropionic acid (TMSP-d_4_) (δ_H_ = 0.00 ppm, δ_C_ = 0.0 ppm) or residual CD_3_OD (δ_H_ = 3.31 ppm, δ_C_ = 49.0 ppm).

## 4. Conclusions

This is the first report on a series of novel betacyanins not reported in any plant and especially not in the *B. alba* varieties. The acylation with the discovered nitrogenous acyl substituents was established by high-resolution mass spectrometry and is a new phenomenon not only observed in the betacyanin group of pigments so far but also, to the best of our knowledge, in other polyphenolic compounds. The NMR structural experiments resulted in identification of the novel betacyanins, 6’*O*-*E*-caffeoyl-gomphrenin (malabarin) and 6’*O*-*E*-sinapoyl-gomphrenin (gandolin), as well as further confirming the presence of 6’-*O*-*E*-4-coumaroyl-gomphrenin (globosin) and 6’-*O*-*E*-feruloyl-gomphrenin (basellin) in *B. alba* matured fruit extracts by two-dimensional NMR techniques.

In this respect, further investigations of *B. alba* fruits as well as their processed products should significantly enhance our knowledge about the bioactivity of betacyanins and especially gomphrenins. Considering that the acylated gomphrenins are found together at relatively high concentrations in *B. alba* L. fruits, this makes this plant material an extremely valuable bioactive source of betacyanins for future food applications.

## Figures and Tables

**Table 1:** Chromatographic, spectrophotometric and mass spectrometric data of the analyzed betacyanins detected in *B. alba* and *B. alba* var. ‘*Rubra*’ fruit juices.

No.	Compound	R_t_ (min)	λ_max_ (nm)	*m*/*z* [M+H]^+^	*m*/*z* LC-ESI-(+)-MS/MS
**1**	betanidin 5-*O*-*β*-sophoroside (melocactin)	5.5	537	713	551; 389
**2**	(hexosyl)-(hexosyl)-betanidin ^a^	5.7	- ^b^	713	551; 389
**3**	(hexosyl)-betanidin ^a^	5.7	539	551	389
**1** **’**	isobetanidin 5-*O*-*β*-sophoroside (isomelocactin)	5.9	537	713	551; 389
**3** **’**	(hexosyl)-isobetanidin ^a^	6.2	539	551	389
**2** **’**	(hexosyl)-(hexosyl)-isobetanidin ^a^	6.3	537	713	551; 389
**4**	betanidin 5-*O*-*β*-glucoside (betanin)	6.6	535	551	389
**5**	betanidin 6-*O*-*β*-sophoroside (bougainvillein-v)	6.8	542	713	551; 389
**6**	(hexosyl)-(hexosyl)-betanidin ^a^	6.9	536	713	551; 389
**4** **’**	Isobetanidin 5-*O*-*β*-glucoside (isobetanin)	7.3	535	551	389
**5** **’**	isobetanidin 6-*O*-*β*-sophoroside (isobougainvillein-v)	7.3	542	713	551; 389
**6** **’**	(hexosyl)-(hexosyl)-isobetanidin ^a^	7.5	536	713	551; 389
**7**	betanidin 6-*O*-*β*-glucoside (gomphrenin)	8.2	537	551	389
**7** **’**	isobetanidin 6-*O*-*β*-glucoside (isogomphrenin)	8.7	537	551	389
**8a**	3’-*O*-malonyl-gomphrenin ^a^	8.7	536	637	593; 551; 389
**8b**	4’-*O*-malonyl-gomphrenin ^a^	9.1	538	637	593; 551; 389
**8a** **’**	3’-*O*-malonyl-isogomphrenin ^a^	9.5	538	637	593; 551; 389
**8c**	6’-*O*-malonyl-gomphrenin ^a^	9.7	537	637	593; 551; 389
**8b** **’**	4’-*O*-malonyl-isogomphrenin ^a^	10.0	538	637	593; 551; 389
**8c** **’**	6’-*O*-malonyl-isogomphrenin ^a^	10.3	537	637	593; 551; 389
**9**	C_9_H_8_NO_4_-gomphrenin ^a^	10.4	543	744	700; 656; 612; 568; 551; 389
**10a**	(3’’-hydroxy-butyryl)-(hexosyl)-betanidin ^a^	10.7	537	637	551; 389
**11**	C_10_H_10_NO_5_-gomphrenin ^a^	10.8	542	774	742; 389
**12**	(hexosyl)-(coumaroyl-hexosyl)-betanidin ^a^	11.0	543	859	697; 551; 389
**10b**	(3’’-hydroxy-butyryl)-(hexosyl)-isobetanidin ^a^	11.0	537	637	551; 389
**9** **’**	C_9_H_8_NO_4_-isogomphrenin ^a^	11.2	543	744	700; 656; 612; 568; 551; 389
**10c**	3’’-hydroxy-butyryl-gomphrenin ^a^	11.4	538	637	551; 389
**12** **’**	(hexosyl)-(coumaroyl-hexosyl)-isobetanidin ^a^	11.5	543	859	697; 551; 389
**11** **’**	C_10_H_10_NO_5_-isogomphrenin ^a^	11.8	- ^b^	774	-
**10d**	3’’-hydroxy-butyryl-isogomphrenin ^a^	11.9	538	637	551; 389
**13**	C_8_H_6_NO_3_-gomphrenin ^a^	12.6	542	714	670; 626; 582; 551; 538; 389
**14**	C_7_H_8_NO_2_-gomphrenin ^a^	12.7	538	688	644; 600; 389
**13** **’**	C_8_H_6_NO_3_-isogomphrenin ^a^	12.9	542	714	670; 626; 582; 551; 538; 389
**15**	6’*O*-*E*-caffeoyl-gomphrenin (malabarin)	12.9	545	713	551; 389
**14** **’**	C_7_H_8_NO_2_-isogomphrenin ^a^	13.1	538	688	644; 600; 389
**16**	*Z*-isomer of globosin ^a^	13.4	544	697	653; 551; 389
**17**	*Z*-isomer of basellin ^a^	13.4	544	727	551; 389
**15** **’**	6’*O*-*E*-caffeoyl-isogomphrenin (isomalabarin)	13.6	545	713	551; 389
**18**	(hexosyl)-(coumaroyl-hexosyl)-betanidin ^a^	13.7	543	859	697; 551; 389
**16** **’**	*Z*-isomer of isoglobosin ^a^	13.8	544	697	653; 551; 389
**17** **’**	*Z*-isomer of isobasellin ^a^	13.8	545	727	551; 389
**18** **’**	(hexosyl)-(coumaroyl-hexosyl)-isobetanidin ^a^	13.9	543	859	697; 551; 389
**19**	6’*O*-*E*-sinapoyl-gomphrenin (gandolin)	14.3	544	757	713; 551; 389
**20**	6’*O*-*E*-4-coumaroyl-gomphrenin (globosin)	14.5	544	697	653; 551; 389
**21**	6’*O*-*E*-feruloyl-gomphrenin (basellin)	14.5	545	727	551; 389
**20** **’**	6’*O*-*E*-4-coumaroyl-isogomphrenin (isoglobosin)	15.3	544	697	653; 551; 389
**19** **’**	6’*O*-*E*-sinapoyl-isogomphrenin (isogandolin)	15.5	544	757	551; 389
**21** **’**	6’*O*-*E*-feruloyl-isogomphrenin (isobasellin)	15.5	545	727	551; 389

^a^ Tentatively identified; ^b^ Due to coelution with impurities, the λ_max_ could not be observed.

**Table 2:** High-resolution mass spectrometric data (obtained by the Orbitrap system) in identification of novel betacyanins possessing non-nitrogenous substituents present in *B. alba* and *B. alba* var. ‘Rubra’ fruit juices.

No.	Compound ^a^	Molecular Formula	[M+H]^+^ Observed	[M+H]^+^ Predicted	Error (mDa)	Error (ppm)	MS^2^ Ions
**2**	hex-hex-Bd	C_30_H_37_N_2_O_18_	713.2030	713.2036	−0.6	−0.84	551.1502 (-hex); 389.0975 (-hex-hex)
**3**	hex-Bd	C_24_H_27_N_2_O_13_	551.1505	551.1508	−0.3	−0.54	389.0973 (-hex)
**6**	hex-hex-Bd	C_30_H_37_N_2_O_18_	713.2033	713.2036	−0.3	−0.42	695.1904 (-H_2_O); 551.1502 (-hex); 389.0975 (-hex-hex)
**8a**	3’-mal-Gp	C_27_H_29_N_2_O_16_	637.1507	637.1512	−0.5	−0.78	619.1409 (-H_2_O); 593.1608 (-CO_2_); 551.1500 (-mal); 389.0976 (-mal-glc)
**8b**	4’-mal-Gp	C_27_H_29_N_2_O_16_	637.1508	637.1512	−0.4	−0.63	619.1413 (-H_2_O); 593.1611 (-CO_2_); 551.1503 (-mal); 389.0973 (-mal-glc)
**8c**	6’-mal-Gp	C_27_H_29_N_2_O_16_	637.1513	637.1512	0.1	0.16	619.1402 (-H_2_O); 593.1603 (-CO_2_); 551.1497 (-mal); 389.0977 (-mal-glc)
**10a**	(3’’-OH-but)-hex-Bd	C_28_H_33_N_2_O_15_	637.1870	637.1876	−0.6	−0.94	593.1978 (-CO_2_); 551.1498 (-3-OH-but); 389.0968 (-3-OH-but-glc)
**10b**	(3’’-OH-but)-hex-Bd	C_28_H_33_N_2_O_15_	637.1869	637.1876	−0.7	−1.10	593.1973 (-CO_2_); 551.1492 (-3-OH-but); 389.0977 (-3-OH-but-glc)
**10c**	3’’-OH-but-Gp	C_28_H_33_N_2_O_15_	637.1875	637.1876	−0.1	−0.16	593.1982 (-CO_2_); 551.1495 (-3-OH-but); 389.0971 (-3-OH-but-glc)
**10d**	3’’-OH-but-isoGp	C_28_H_33_N_2_O_15_	637.1871	637.1876	−0.5	−0.78	593.1976 (-CO_2_); 551.1497 (-3-OH-but); 389.0973 (-3-OH-but-glc)
**12**	hex-(coum)-hex-Gp	C_39_H_43_N_2_O_20_	859.2398	859.2404	−0.6	−0.70	815.2499 (-CO_2_); 697.1868 (-hex); 653.1984 (-hex, -CO_2_); 551.1502 (-coum-hex); 389.0979 (-coum-hex-hex)
**15**	caff-Gp	C_33_H_33_N_2_O_16_	713.1820	713.1825	−0.5	−0.70	669.1913 (-CO_2_); 625.2020 (-2CO_2_); 551.1503 (-caff); 389.0973 (-caff-glc)
**16**	*Z*-coum-Gp	C_33_H_33_N_2_O_15_	697.1874	697.1876	−0.2	−0.29	653.1985 (-CO_2_); 609.2075 (-2CO_2_); 551.1499 (-*Z*-coum); 389.0973 (-*Z*-coum-glc)
**17**	*Z*-fer-Gp	C_34_H_35_N_2_O_16_	727.1982	727.1981	0.1	0.14	683.2061 (-CO_2_); 551.1495 (-*Z*-fer); 389.0971 (-*Z*-fer-glc)
**18**	hex-(coum)-hex-Gp	C_39_H_43_N_2_O_20_	859.2407	859.2404	0.3	0.35	841.2291 (-H_2_O); 713.2050 (-coum); 697.1877 (-hex); 551.1503 (-coum-hex); 389.0972 (-coum-hex-hex)
**19**	sin-Gp	C_35_H_37_N_2_O_17_	757.2083	757.2087	−0.4	−0.53	713.2178 (-CO_2_); 669.2291 (-2CO_2_); 551.1502 (-sin); 389.0973(-sin-glc)

^a^ Abbreviations: hex—hexosyl; mal—malonyl; but—butyryl; caff—caffeoyl; coum—coumaroyl; fer—feruloyl; sin—sinapoyl; glc—glucosyl; Bd—betanidin; Gp—gomphrenin.

**Table 3:** High-resolution mass spectrometric data obtained by analysis of *B. alba* and *B. alba* var. ‘Rubra’ *fruit juices* by the Orbitrap system indicating the presence of novel natural betacyanins acylated with nitrogenous substituents.

No.	Compound ^a^	Molecular Formula	[M+H]^+^ Observed	[M+H]^+^ Predicted	Error (mDa)	Error (ppm)
**9**	[C_9_H_8_NO_4_-Gp +H]^+^	C_33_H3_4_N_3_O_17_	744.1878	744.1883	−0.5	−0.67
	nl: -CO_2_	C_32_H_34_N_3_O_15_	700.1988	700.1984	0.4	0.51
	nl: -2CO_2_	C_31_H_34_N_3_O_13_	656.2078	656.2086	−0.8	−1.24
	nl: -3CO_2_	C_30_H_34_N_3_O_11_	612.2181	612.2188	−0.7	−1.12
	nl: -4CO_2_	C_29_H_34_N_3_O_9_	568.2274	568.2290	−1.6	−2.74
	nl: -C_9_H_8_NO_4_	C_24_H_27_N_2_O_13_	551.1529	551.1508	2.1	3.81
	nl: -C_9_H_8_NO_4_-glc	C_18_H_17_N_2_O_8_	389.0973	389.0979	−0.6	−1.65
**11**	[C_10_H_10_NO_5_-Gp +H]^+^	C_34_H_36_N_3_O_18_	774.1975	774.1988	−1.3	−1.68
	nl: -CH_3_OH	C_33_H_32_N_3_O_17_	742.1715	742.1726	−1.1	−1.48
	nl: -CH_3_OH; -CO_2_	C_32_H_32_N_3_O_15_	698.1834	698.1828	0.6	0.86
	nl: -CH_3_OH; -2CO_2_	C_31_H_32_N_3_O_13_	654.1935	654.1930	0.5	0.76
	nl: -CH_3_OH; -3CO_2_	C_30_H_32_N_3_O_11_	610.2034	610.2031	0.3	0.49
	nl: -CH_3_OH; -4CO_2_	C_29_H_32_N_3_O_9_	566.2142	566.2133	0.9	1.59
	nl: -C_10_H_10_NO_5_-glc	C_18_H_17_N_2_O_8_	389.0973	389.0979	−0.6	−1.54
**13**	[C_8_H_6_NO_3_-Gp +H]^+^	C_32_H_32_N_3_O_16_	714.1776	714.1777	−0.1	−0.14
	nl: -CO_2_	C_31_H_32_N_3_O_14_	670.1875	670.1879	−0.4	−0.57
	nl: -2CO_2_	C_30_H_32_N_3_O_12_	626.1965	626.1981	−1.5	−2.48
	nl: -3CO_2_	C_29_H_32_N_3_O_10_	582.2072	582.2082	−1.0	−1.75
	nl: -C_8_H_6_NO_3_	C_24_H_27_N_2_O_13_	551.1495	551.1508	−1.3	−2.36
	nl: -4CO_2_	C_28_H_32_N_3_O_8_	538.2166	538.2184	−1.8	−3.33
	nl: -C_8_H_6_NO_3_-glc	C_18_H_17_N_2_O_8_	389.0974	389.0979	−0.5	−1.39
**14**	[C_7_H_8_NO_2_-Gp +H]^+^	C_31_H_34_N_3_O_15_	688.1983	688.1984	−0.1	−0.15
	nl: -CO_2_	C_30_H_34_N_3_O_13_	644.2069	644.2086	−1.7	−2.66
	nl: -2CO_2_	C_29_H_34_N_3_O_11_	600.2170	600.2188	−1.8	−2.97
	nl: -3CO_2_	C_28_H_34_N_3_O_9_	556.2283	556.2290	−0.7	−1.18
	nl: -C_7_H_8_NO_2_-glc	C_18_H_17_N_2_O_8_	389.0978	389.0979	−0.1	−0.36

^a^ Abbreviations: nl (neutral loss); glc—glucosyl; Gp—gomphrenin.

**Table 4:** The NMR data obtained in D_2_O (**15**, **20** and **21**) and CD_3_OD/d-TFA (**19**) for the principal acylated betacyanins isolated from *Basella alba* L. fruits. The ^1^H and ^13^C spectra of the pigments are presented in Figures S1–S8.

	6’-*O*-*E*-caffeoyl-gomphrenin 15		6’-*O*-*E*-sinapoyl-gomphrenin 19		6’-*O*-*E*-4-coumaroyl-gomphrenin 20		6’-*O*-*E*-feruloyl-gomphrenin 21	
No.	^1^H NMR ^a^	^13^C ^b,c^	^1^H NMR ^a^	^13^C ^b,c^	^1^H NMR ^a^	^13^C ^b,c^	^1^H NMR ^a^	^13^C ^b,c^
**2**	3.88, *bm*	67.5	4.72, *dd*, 3.2; 10.0	64.5	3.85, *bdd*,	65.2	3.79, *dd*, 3.3; 10.2	64.7
**3a/b**	3.31, *bm* 3.04 (overlap)	35.9	3.38, *dd*, 10.2; 16.3 3.26, *dd*, 16.8; 3.0	33.4	3.34, *dd*, 10.4; 16.6 3.13, *dd*, 16.3; 3.7	33.9	3.29, *dd*, 10.3; 16.1 3.12, *dd*, 16.6; 3.2	34.0
**4**	6.76, *s*	116.2	6.87, *s*	113.8	6.80 (overlap)	114.1	6.79, *s*	113.9
**5**		145.8		146.9		144.7		144.8
**6**		146.8		149.6		145.7		145.8
**7**	6.84, *s*	100.9	7.42, *s*	103.0	6.80, *s*	98.9	6.77 (overlap)	98.6
**8**		137.1		134.4		135.1		135.0
**9**		129.3		129.1		127.2		126.7
**10**		178.2		171.1		175.4		174.7
**11**	7.88, *d*, 12.5	144.4	8.58, *d*, 12.3	147.0	8.05, *d*, 12.5	143.0	8.03, *d*, 12.4	143.3
**12**	5.27, *d*, 12.3	109.5	5.93, *d*, 12.2	110.5	5.38, *d*, 12.4	108.4	5.39, *d*, 12.3	108.4
**13**		164.4		163.0		162.4		162.0
**14a/b**	3.08 (overlap) 3.02 (overlap)	29.9	3.67, *dd*, 17.5; 5.1 3.20, *bs*	27.7	3.22, *bm* 3.04, *bm*	27.9	3.20, *bm* 3.04, *bm*	27.9
**15**	4.28, *bm*	56.4	4.53, *bt*, 7.2	53.2	4.41, *bt*, 9.2	53.9	4.47, *bt*, 6.8	53.5
**17**		158.4		150.2		156.0		155.0
**18**	6.04, *bs*	107.5	6.33, *bs*	106.6	6.13, *bs*	105.6	6.11, *s*	105.6
**19**		178.5		172.9		175.8		175.0
**20**		170.4		167.4		169.3		167.8
**1’**	5.01, *d*, 7.1 ^d^	100.6	4.96, *d*, 7.7	102.1	5.11, *d*, 6.7 ^d^	98.6	4.85, *d*, 6.9 ^d^	98.4
**2’**	3.64 (overlap)	78.3	3.53 (overlap)	77.4	3.61 (overlap)	76.4	3.61(overlap)	76.4
**3’**	3.63 (overlap)	75.3	3.58 (overlap)	74.4	3.66 (overlap)	73.5	3.66 (overlap)	73.3
**4’**	3.43 (overlap)	73.8	3.45 (overlap)	71.9	3.44 (overlap)	71.8	3.44 (overlap)	71.9
**5’**	3.96 (overlap)	76.8	3.84 (overlap)	75.2	3.96 (overlap)	75.1	3.97 (overlap)	74.6
**6’a/b**	4.49, *dd*, 11.9; 4.9 4.37, *dd*, 12.1; 2.2	66.3	4.68, *dd*, 11.9; 5.3 4.42, *dd*, 11.8; 2.5	64.2	4.49 (overlap) 4.48 (overlap)	64.5	4.54, *dd*, 12.0; 5.1 4.34, *dd*, 11.7; 2.1	64.4
**1″**		128.9		126.4		126.9		127.3
**2″**	6.79, *bd*	118.8	6.69, *s*	106.9	7.09 (overlap)	131.3	6.78 (overlap)	111.3
**3″**		146.6		149.6	6.86 (overlap)	116.7		148.5
**4″**		149.7		139.7		159.5		149.0
**5″**	6.69, *bd*	117.8		149.6	6.86 (overlap)	116.7	6.86, *d*, 8.0	116.2
**6″**	6.58, *bdd*	125.4	6.69, *s*	106.9	7.09 (overlap)	131.3	6.57, *bdd*, 7.5	124.4
**7″**	7.01, *d*, 15.1	148.2	7.39, *d*, 16.4	147.3	7.11 (overlap)	146.6	7.01, *d*, 16.4	146.6
**8″**	6.03, *d*, 15.6	117.0	6.47, *d*, 15.8	116.0	6.05, *d*, 15.9	114.8	6.08, *d*, 15.9	115.0
**9″**		171.4		169.3		169.7		169.4
**10″**			3.81, *s*	56.8			3.70, *s*	56.7
**11″**			3.81, *s*	56.8				

^a 1^H NMR *δ* (ppm), mult, *J* (Hz); ^b 13^C NMR *δ* (ppm); ^c 13^C chemical shifts were derived from HSQC, HMBC and ^13^C NMR; ^d^ obtained in a new CD_3_OD/d-TFA soln.

**Table 5:** Total contents and relative concentrations of betacyanins (15*S*) and their isoforms (15*R*) determined in *B. alba* and *B. alba* var. ‘Rubra’ *fruit juices* by LC-DAD-MS measurements.

No.		Relative Betacyanin Concentration (%) ± SD ^a^							
	Compound/	*B. alba*				*B. alba* var. ‘Rubra’			
	/Abbreviation ^c^	Forms	15*S*	Forms	15*R*	Forms	15*S*	Forms	15*R*
**1**	melocactin	0.21	±0.027	0.08	±0.011	0.26	±0.041	0.12	±0.021
**2**	hex-hex-Bd	0.31	±0.039	0.11	±0.018	0.44	±0.071	0.15	±0.020
**3**	hex-Bd	3.7	±0.55	0.31	±0.048	17.5	±2.8	0.48	±0.074
**4**	betanin	0.37	±0.049	0.14	±0.020	0.44	±0.071	0.14	±0.020
**5**	bougainvillein-v	1.4	±0.20	1.1	±0.17	1.9	±0.27	1.5	±0.24
**6**	hex-hex-Bd	0.25	±0.033	0.061	±0.0090	0.44	±0.063	0.13	±0.017
**7**	gomphrenin	39.7	±2.8	13.7	±0.97	43.9	±2.2	13.2	±0.81
**8a**	3’-mal-Gp	0.18	±0.023			0.02	±0.0034		
**8b**	4’-mal-Gp	0.046	±0.0071			0.066	±0.010		
**8c**	6’-mal-Gp	3.9	±0.53	0.46	±0.071	0.79	±0.11	0.12	±0.018
**9**	C_9_H_8_NO_4_-Gp	2.0	±0.29	0.11	±0.018	2.2	±0.37	0.13	±0.021
**10a**	(3’’-OH-but)-hex-Bd	0.24	±0.032			0.066	±0.010		
**11**	C_10_H_10_NO_5_-Gp	0.15	±0.023	0.006	±0.0009	0.19	±0.025	0.018	±0.0027
**12**	hex-(coum-hex)-Bd	0.71	±0.087	0.11	±0.018	0.17	±0.028	0.066	±0.010
**10b**	(3’’-OH-but)-hex-Bd	0.63	±0.099			0.088	±0.013		
**10c/d**	(3’’-OH-but)-Gp	4.4	±0.62	0.46	±0.074	0.61	±0.099	0.19	±0.023
**13**	C_8_H_6_NO_3_-Gp	0.49	±0.064	0.16	±0.16	0.35	±0.053	0.69	±0.10
**14**	C_7_H_8_NO_2_-Gp	2.3	±0.34	0.37	±0.059	0.70	±0.11	0.15	±0.019
**15**	caff-Gp	2.4	±0.31	0.42	±0.067	1.5	±0.22	0.26	±0.041
**16**	*Z*-coum-Gp	0.15	±0.023	0.052	±0.0085	0.15	±0.022	0.044	±0.0065
**17**	*Z*-fer-Gp	0.20	±0.027	0.015	±0.0026	0.64	±0.11	0.11	±0.014
**18**	hex-(coum-hex)-Bd	0.33	±0.038	0.24	±0.037	0.23	±0.035	0.17	±0.020
**19**	sin-Gp	0.46	±0.058	0.18	±0.026	0.57	±0.095	0.20	±0.027
**20**	coum-Gp	12.9	±1.8	1.8	±0.31	6.6	±1.1	0.82	±0.14
**21**	fer-Gp	2.1	±0.28	0.61	±0.098	0.21	±0.19	1.2	±0.035
	Total pigment: content [mg/100 g] ^b^			42.0	±0.25			86.6	±0.67

^a^ Relative concentrations were expressed as percentage of the total peak area. Average of three measurements. ^b^ In betanin equivalents.^c^ Abbreviations: hex—hexosyl; mal—malonyl; but—butyryl; caff—caffeoyl; coum—coumaroyl; fer—feruloyl; sin—sinapoyl; Bd—betanidin; Gp—gomphrenin.

## Data Availability

Not applicable.

## Supplementary Materials

The following supporting information can be downloaded at: https://www.mdpi.com/article/10.3390/ijms231911243/s1.
