# Supplementary material for: Structural Studies on Diverse Betacyanin Classes in Matured Pigment-Rich Fruits of Basella alba L. and Basella alba L. var. ‘Rubra’ (Malabar Spinach)
Source: Int J Mol Sci. 2022 Sep 24;23(19):11243. doi: 10.3390/ijms231911243 (PMC9570114; doi:10.3390/ijms231911243)
Supplement: Supplementary file 1 [file ijms-23-11243-s001.zip › ijms-1900414-supplementary.pdf]

Supplementary Materials

Article

Structural Studies on Diverse Betacyanin Classes in Matured Pigment-Rich Fruits of *Basella alba* L. and *Basella alba* L. var. 'Rubra' (Malabar Spinach)

Katarzyna Sutor-Świeży [1], Michał Antonik [1], Ewa Dziedzic [2], Monika Bieniasz [2], Przemysław Mielczarek [3,4], Łukasz Popenda [5], Karol Pasternak [6], Małgorzata Tyszka-Czochara [7] and Sławomir Wybraniec [1,*]

1 Department C-1, Faculty of Chemical Engineering and Technology, Cracow University of Technology, ul. Warszawska 24, 31-155 Cracow, Poland
2 Faculty of Biotechnology and Horticulture, Agricultural University of Krakow, al. 29 Listopada 54, 31-425 Krakow, Poland
3 Department of Analytical Chemistry and Biochemistry, Faculty of Materials Science and Ceramics, AGH University of Science and Technology, al. Adama Mickiewicza 30, 30-059, Krakow, Poland
4 Laboratory of Proteomics and Mass Spectrometry, Maj Institute of Pharmacology, Polish Academy of Sciences, ul. Smętna 12, 31-343 Krakow, Poland
5 NanoBioMedical Centre, Adam Mickiewicz University, ul. Wszechnicy Piastowskiej 3, 61-614 Poznan, Poland
6 Institute of Bioorganic Chemistry, Polish Academy of Sciences, ul. Noskowskiego 12/14, 61-704 Poznan, Poland
7 Faculty of Pharmacy, Jagiellonian University Medical College, ul. Medyczna 9, 30-688 Krakow, Poland
* Correspondence: slawomir.wybraniec@pk.edu.pl; Tel.: +48-12-628-3074; Fax: +48-12-628-2036

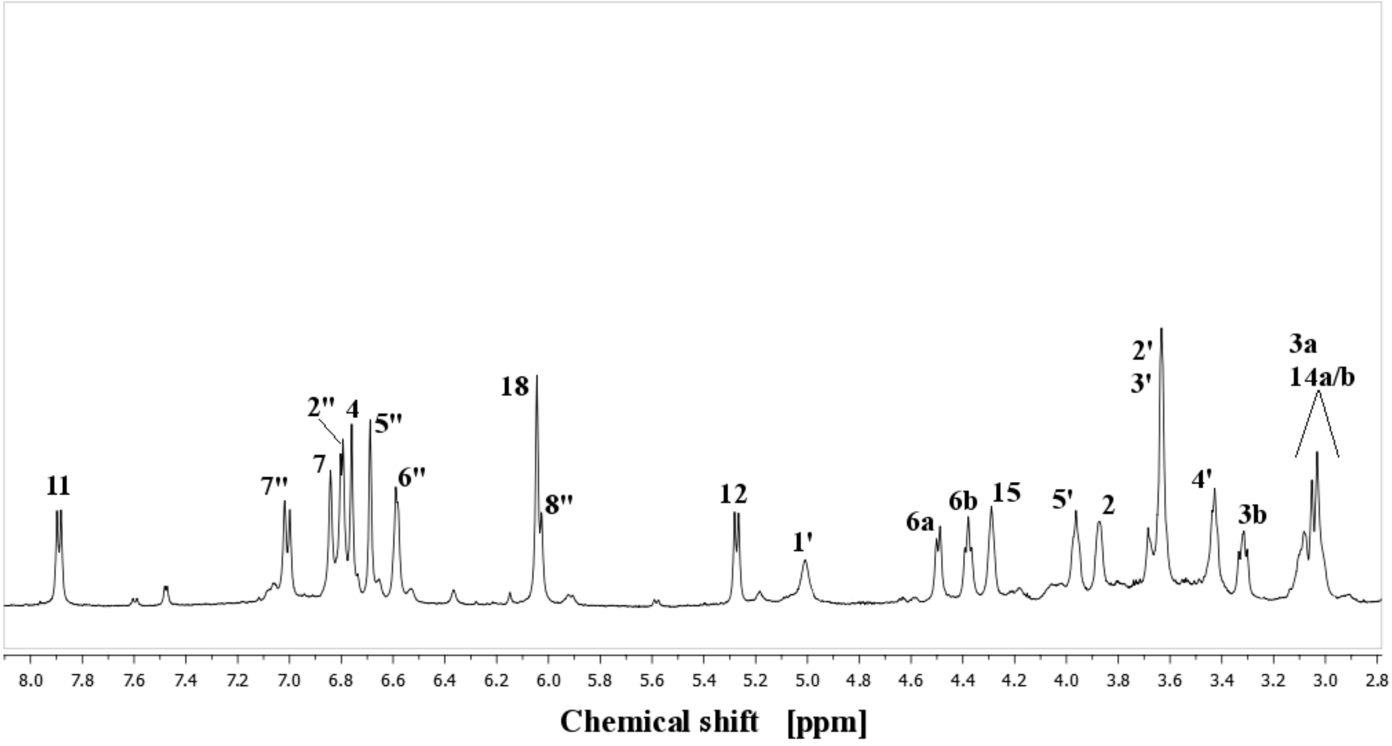

Figure S1. ^{1}H NMR spectrum of 6'-*O*-*E*-caffeoyl-gomphrenin **15** (D_2O, 295 K).

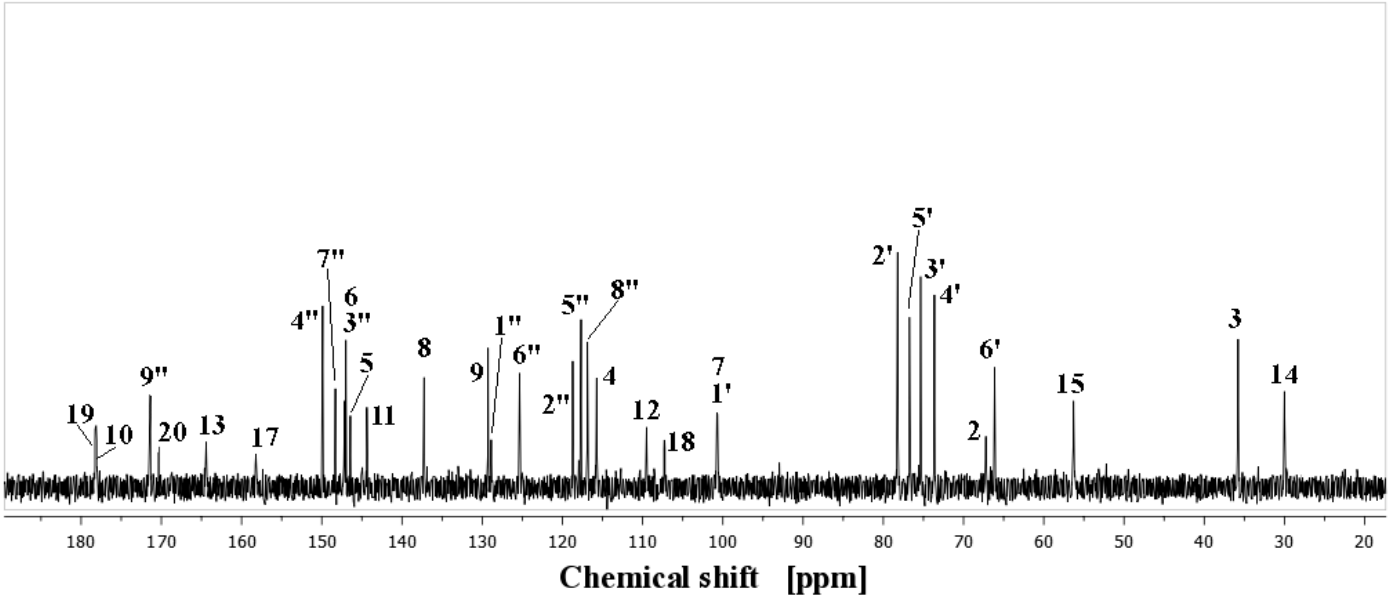

Figure S2. ^{13}C NMR spectrum of 6'-*O*-*E*-caffeoyl-gomphrenin **15** (D_2O, 295 K).

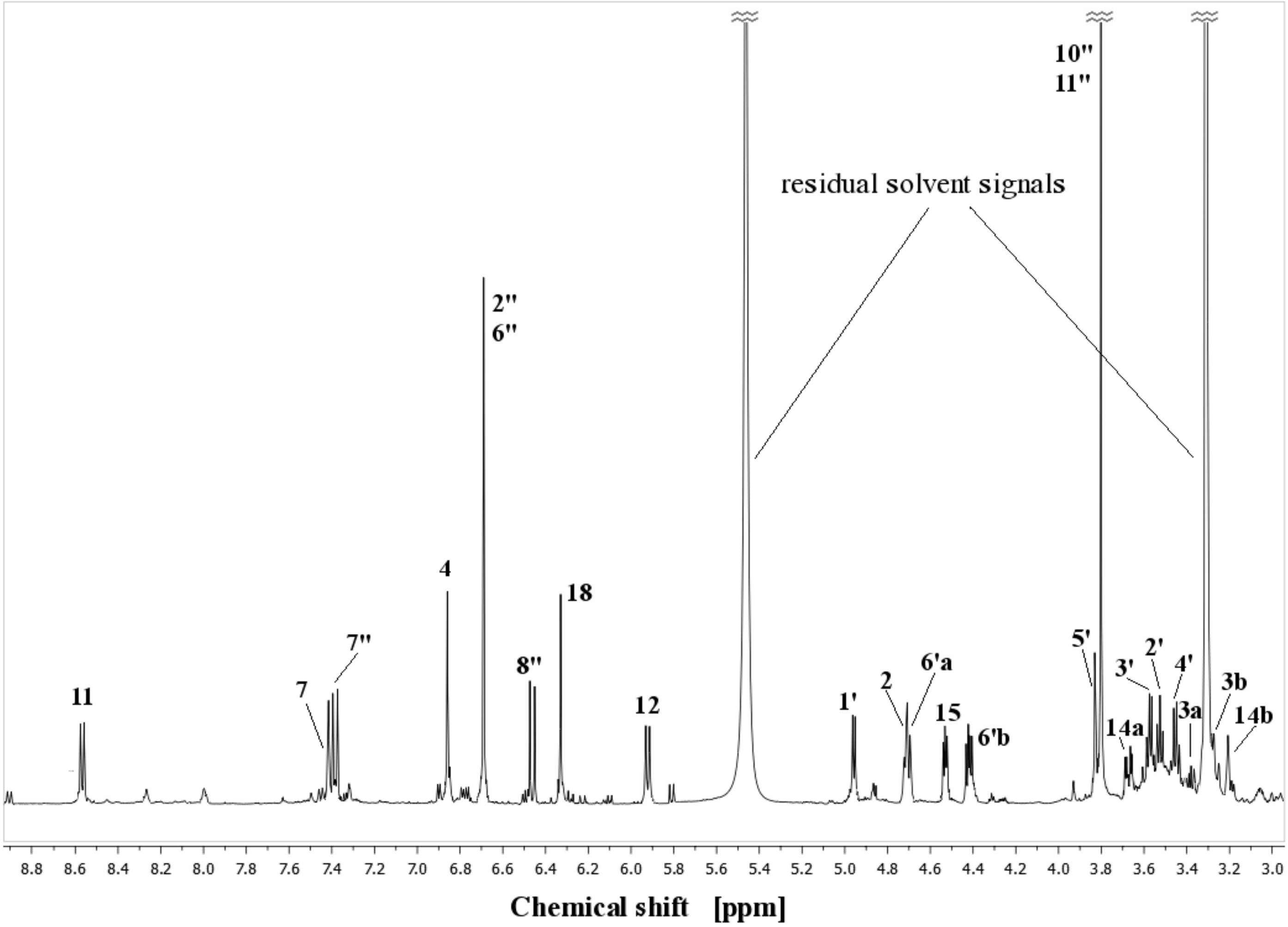

Figure S3. ^{1}H NMR spectrum of 6'-*O*-*E*-sinapoyl-gomphrenin **19** (CD_3OD/d-TFA, 295 K).

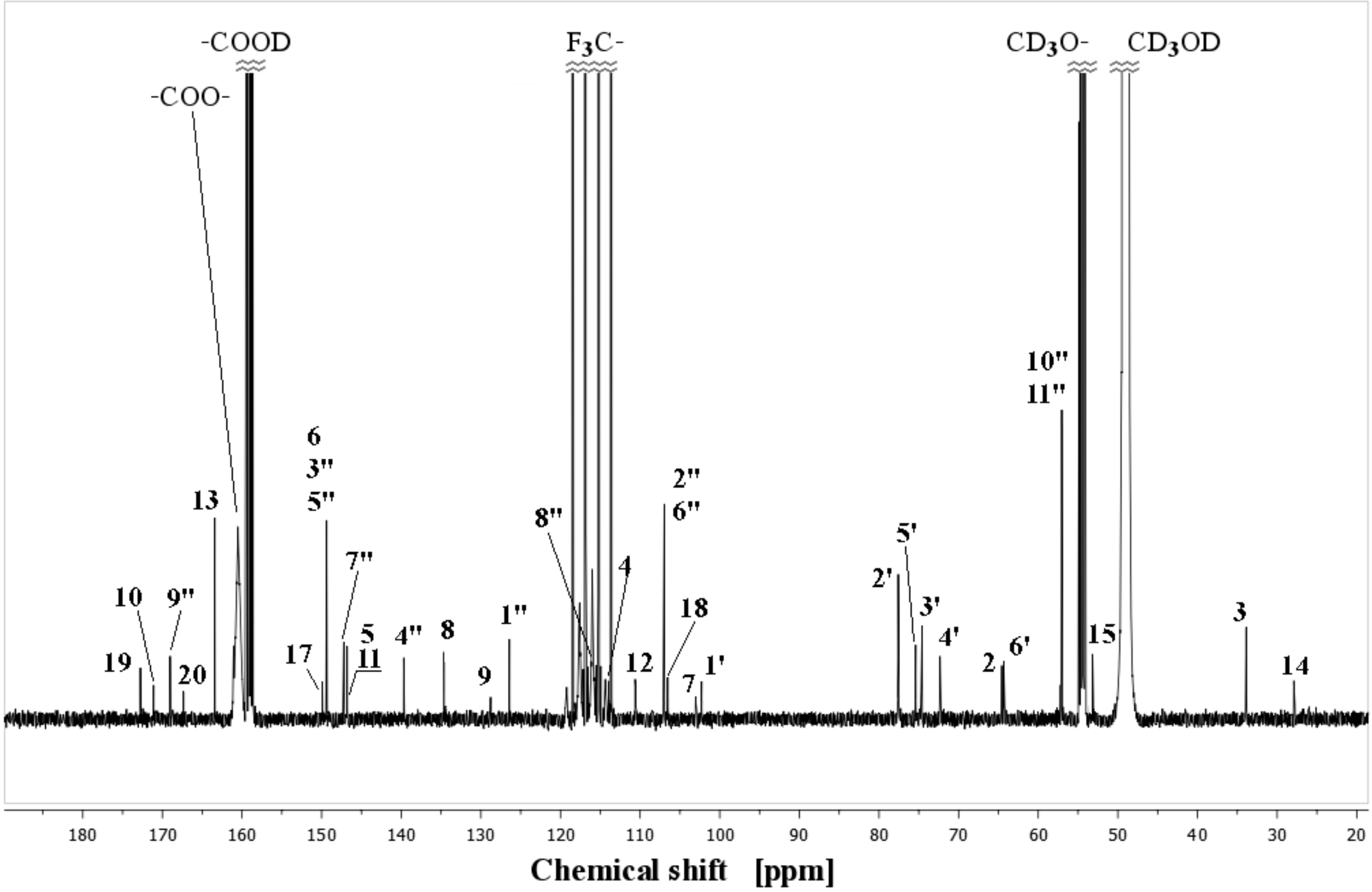

Figure S4. ^{13}C NMR spectrum of 6'-*O*-*E*-sinapoyl-gomphrenin **19** (CD_3OD/d-TFA, 295 K).

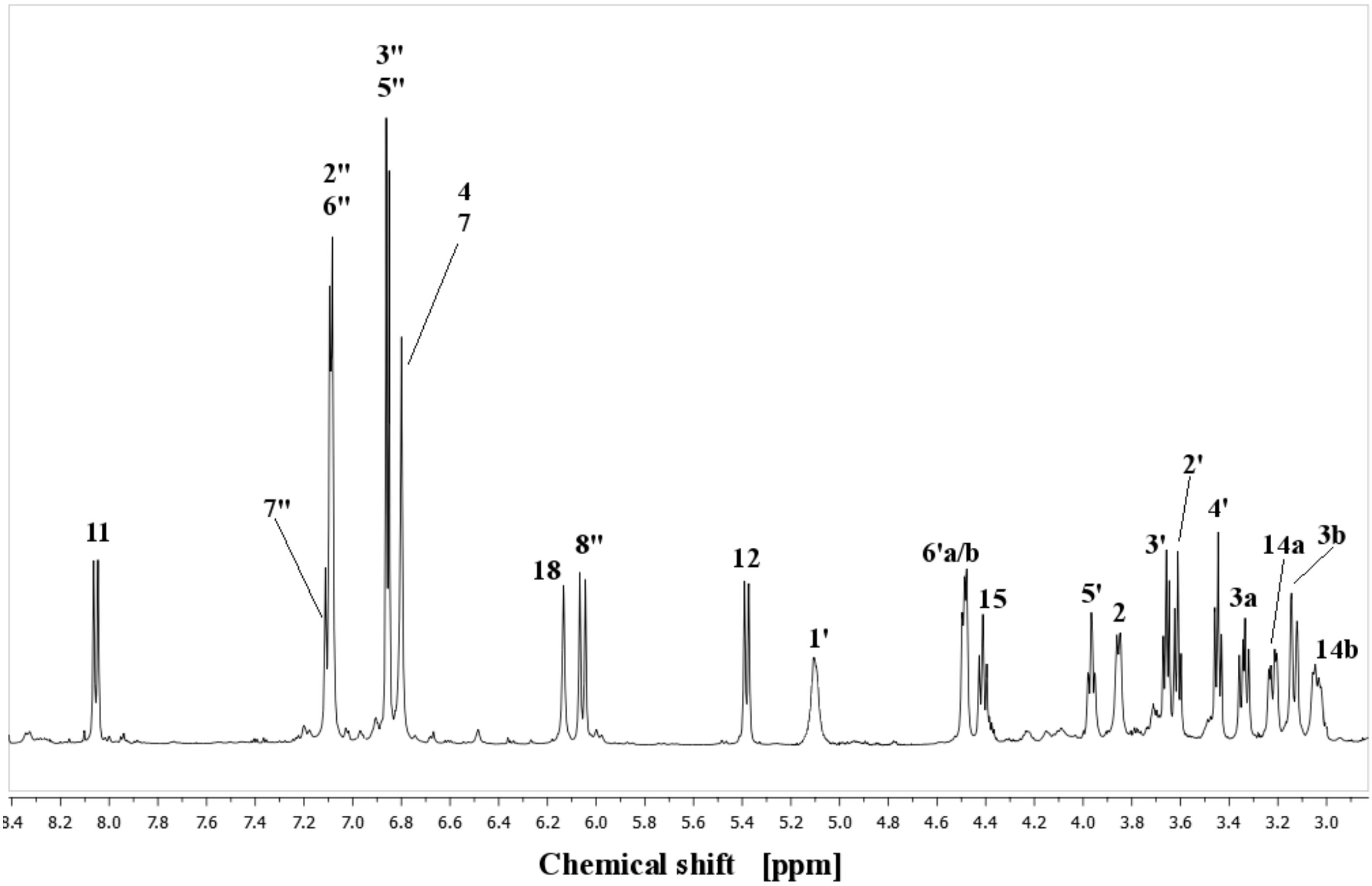

Figure S5. ^{1}H NMR spectrum of 6'-*O*-*E*-4-coumaroyl-gomphrenin **20** (D_2O, 295 K).

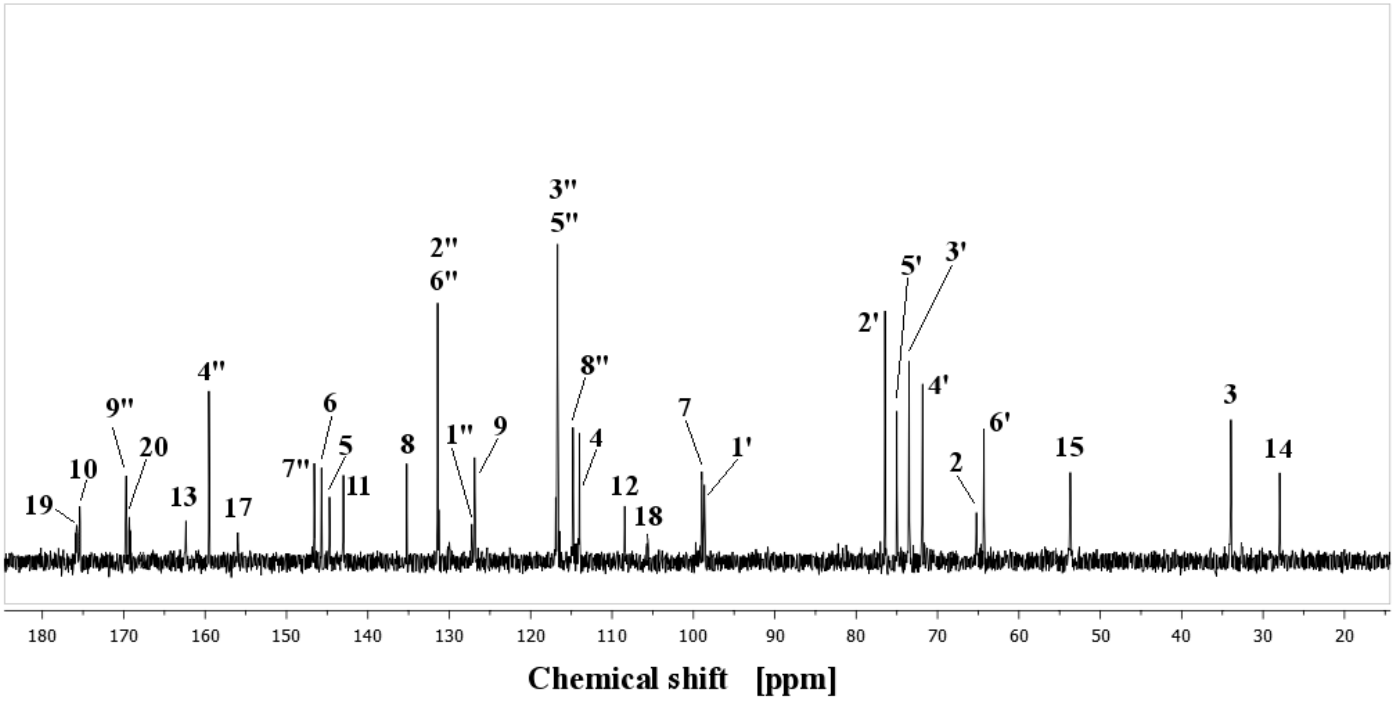

Figure S6. ^{13}C NMR spectrum of 6'-*O*-*E*-4-coumaroyl-gomphrenin **20** (D_2O, 295 K).

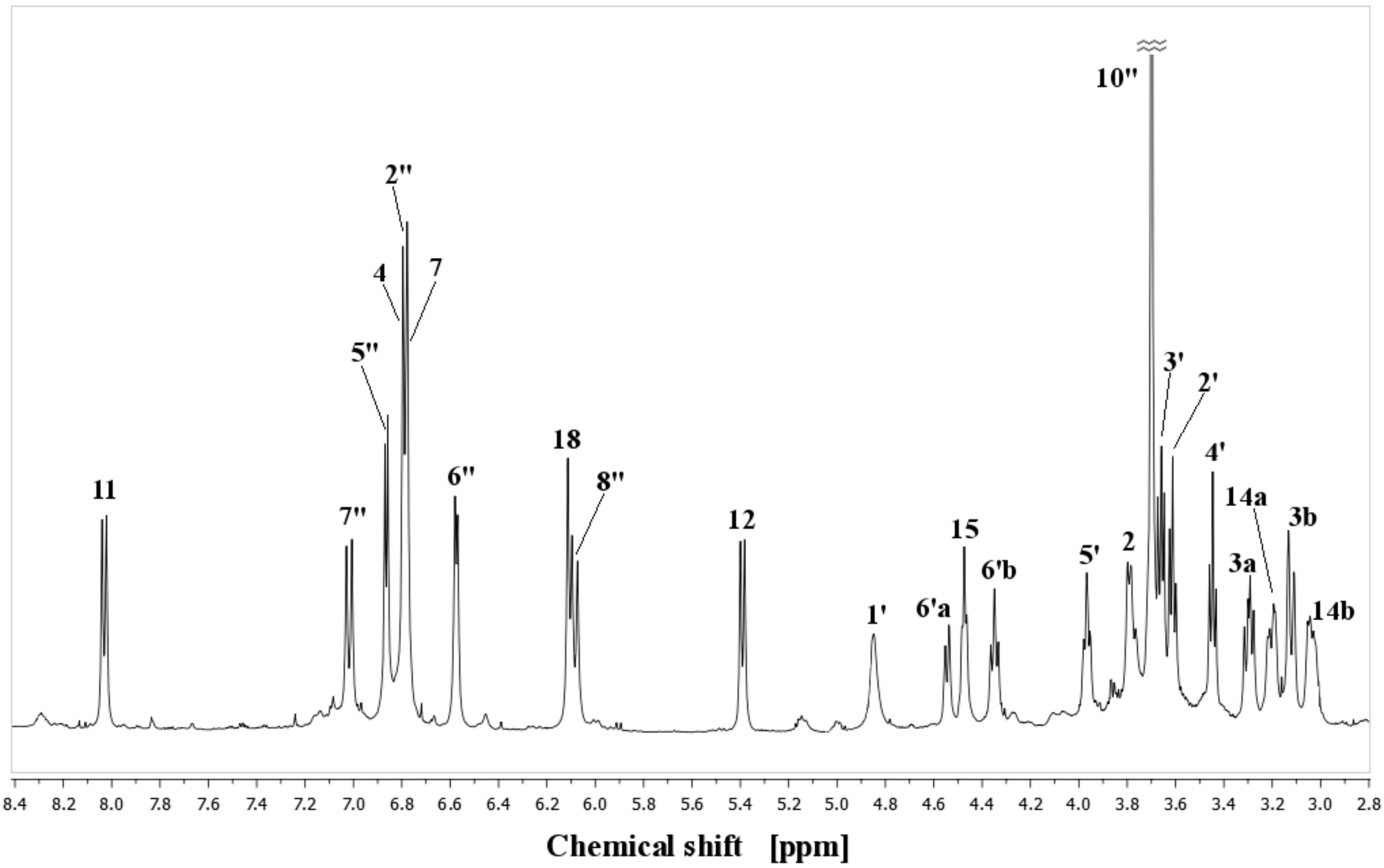

Figure S7. ^{1}H NMR spectrum of 6'-*O*-*E*-feruloyl-gomphrenin **21** (D_2O, 295 K).

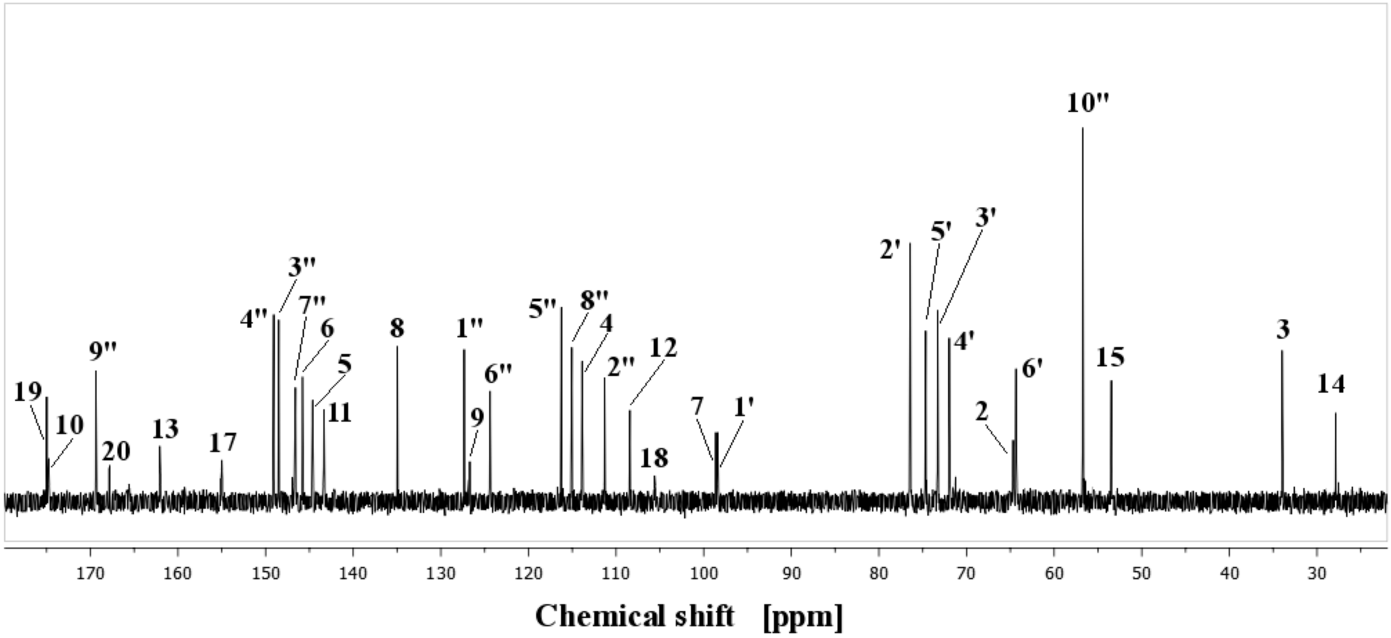

Figure S8. ^{13}C NMR spectrum of 6'-*O*-*E*-feruloyl-gomphrenin **21** (D_2O, 295 K).
